# Design of Novel Mercapto-3-phenylpropanoyl Dipeptides as Dual Angiotensin-Converting Enzyme C–Domain-Selective/Neprilysin Inhibitors

**DOI:** 10.1021/acs.jmedchem.5c00329

**Published:** 2025-04-01

**Authors:** Gyles E. Cozier, Lauren B. Coulson, Charles J. Eyermann, Gregory S. Basarab, Sylva L. Schwager, Kelly Chibale, Edward D. Sturrock, K. Ravi Acharya

**Affiliations:** ± Department of Life Sciences, University of Bath, Claverton Down, Bath BA2 7AY, United Kingdom; † Institute of Infectious Disease and Molecular Medicine, University of Cape Town, Observatory 7925, South Africa; ‡ Department of Integrative Biomedical Sciences, University of Cape Town, Observatory 7925, South Africa; ⊥ Drug Discovery and Development Centre (H3D), University of Cape Town, Rondebosch 7701, South Africa; § Department of Chemistry, University of Cape Town, Rondebosch 7701, South Africa; ∥ South African Medical Research Council Drug Discovery and Development Research Unit, University of Cape Town, Rondebosch 7701, South Africa

## Abstract

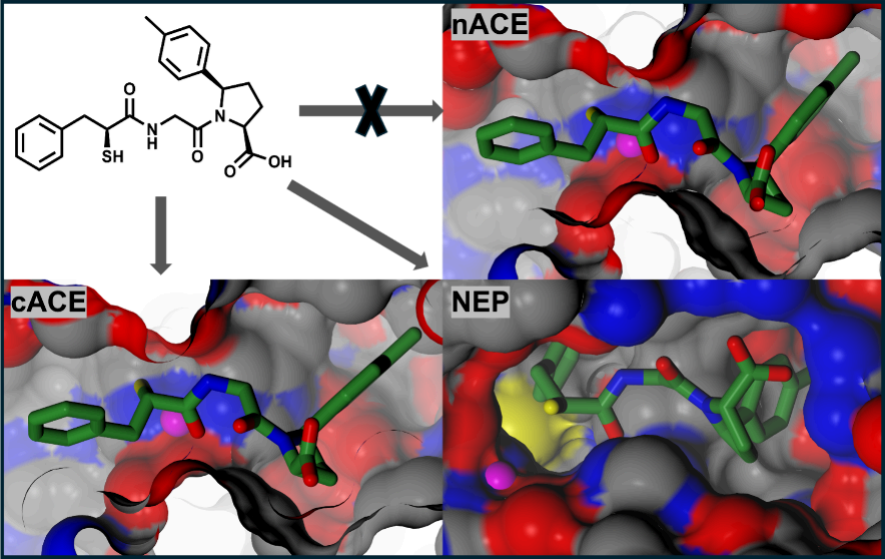

Dual angiotensin-converting enzyme (ACE) and neprilysin (NEP) inhibitors such as omapatrilat showed promise as potent treatments for hypertension but produced adverse effects due to their high affinity for both domains of ACE (nACE and cACE). This led to the search for compounds that retained NEP potency but selectively inhibit cACE, leaving nACE active to degrade other peptides such as bradykinin. Lisinopril-tryptophan (LisW) has previously been reported to have cACE selectivity. Three mercapto-3-phenylpropanoyl inhibitors were synthesized, combining features of omapatrilat and LisW to probe structural characteristics required for potent dual cACE/NEP inhibition. We report the synthesis of these inhibitors, enzyme inhibition data, and high-resolution crystal structures in complex with nACE and cACE. This provides valuable insight into factors driving potency and selectivity and shows that the mercapto-3-phenylpropanoyl backbone is significantly better for NEP potency than a P_1_ carboxylate. Future chemistry efforts could be directed at identifying alternative chemotypes for optimization of cACE/NEP inhibitors.

## Introduction

The renin-angiotensin system (RAS) plays a critical role in blood pressure regulation and fluid and electrolyte balance. Drugs targeting different components of the RAS are widely used for the treatment of hypertension and cardiovascular disease. Since the discovery of captopril in the mid-70s, there have been significant advances in hypertension therapies for patients with cardiovascular disease. However, blockade of the RAS does not always lead to a sufficient reduction in blood pressure, and many patients require combination therapy to achieve their target blood pressure. Alternative peptide–receptor axes, such as the natriuretic peptide, kallikrein-kinin, and endothelin-converting enzyme systems, help to maintain cardiovascular homeostasis, and dual and triple inhibitors of these systems have been developed to provide improved cardiovascular protection and blood pressure control. Dual angiotensin-I-converting enzyme (ACE)/neprilysin (NEP) inhibitors have received much attention as a more effective therapeutic intervention due to the beneficial effects of natriuretic peptides on blood pressure and endothelial function.^1,2^ However, decreased bradykinin hydrolysis and subsequent adverse effects have hampered the development of these drugs. A classic example is the ACE/NEP inhibitor omapatrilat that showed potent antihypertensive effects with beneficial effects on cardiac function heart failure patients.^3,4^ However, the OCTAVE study (Omapatrilat Cardiovascular Treatment Assessment Versus Enalapril) was associated with a 3-fold higher incidence of angioedema than observed for the ACE inhibitor enalapril, which led to failure to receive FDA approval.^5−8^

ACE and NEP are both metallopeptidases that rely on a zinc ion to polarize the substrate carbonyl and assist in the deprotonation of the water nucleophile. ACE is a type I membrane protein that converts the inactive decapeptide angiotensin I (Ang I) to the vasopressor Ang II and inactivates the vasodilator bradykinin.^9,10^ ACE is composed of two catalytically active domains (nACE and cACE) joined by a 15-amino acid linker region.^11^ Despite the two domains sharing 60% sequence similarity, which increases to 89% in the active sites, they differ in their N-linked glycosylation, requirement for chloride ion activation, affinity for inhibitors, and substrate specificity.^12^ cACE is primarily responsible for the production of Ang II,^13^ whereas nACE is highly selective for the antifibrotic peptide *N*-acetyl-Ser-Asp-Lys-Pro (AcSDKP)^14^ and gonadotropin-releasing hormone (GnRH).^15^ Although ACE inhibitors are recommended as a first-choice therapy for hypertension, their use has been associated with adverse effects, such as persistent cough and angioedema as a result of increased levels of bradykinin.^16,17^ NEP is a type II membrane protein that cleaves a broad range of substrates, including natriuretic peptides, Ang I, Ang II, bradykinin, endothelin-1, substance P, and amyloid-β peptide.^18−22^ NEP has large flexible ligand binding site accounting for its broad selectivity, and the prime side is mainly responsible for substrate selectivity and potency.^23,24^

We have developed LisW, a potent inhibitor of the catalytic site of cACE,^25^ which has a bulky tryptophan-like P_2_′ group resulting in its C-selectivity. LisW in combination with the NEP inhibitor sacubitril revealed favorable antihypertensive and cardiovascular effects on Ang II-dependent hypertensive mice, but without decreased bradykinin metabolism.^26^ Using high-resolution crystal structures of LisW in complex with cACE, as well as structures of NEP in complex with omapatrilat and sampatrilat,^27,28^ we designed a series of dual cACE/NEP 1-carboxy-3-phenylpropyl inhibitors based on LisW.^29,30^ These studies revealed important insights into the drivers of NEP and cACE selectivity, including, first, the importance of the P_1_′ group for C-selectivity and the effect of the S_1_′ and S_2_′ subsites’ architecture on P_1_′ and P_2_′ binding. Second, as observed with ACE, there was an interaction between the NEP S_1_′ and S_2_′ subsites wherein when the S_2_′ subsite is occupied by a bulky group, and the Trp693 side chain will move toward the S_1_′ subsite, constricting this pocket.^29^ Thus, a large group can be accommodated by the NEP S_1_′ or S_2_′ subsites, but not by both. Third, a LisW analogue with a P_1_ carboxylate zinc binding group (ZBG) and a phenylpropyl P_2_′ group (AD013) had a reduced affinity for cACE and NEP compared to similar zinc mercapto ZBG analogues with the same P_1_′ and P_2_′ groups. Interestingly, small molecules are accommodated in different orientations by the ACE and NEP catalytic sites. Thus, the phenyl group of omapatrilat binds to the S_1_ subsite of cACE and nACE and the S_1_′ pocket of NEP. For inhibitors without P_1_′ groups suitable for binding to the S_1_′ pocket of NEP, inhibitors with a 2-mercapto-3-phenylpropyl *N*-terminus are more versatile in terms of binding to NEP in this alternative orientation.

In this study, we have designed three novel mercapto LisW analogues to investigate the structural basis for selective and potent cACE/NEP inhibition. This has identified that a thiol-carbonyl ZBG consistently increases the affinity for NEP and the ACE domains compared to the P_1_ carboxylate of LisW-like inhibitors. In addition, two of the novel inhibitors are moderately cACE-selective, indicating that careful choice of inhibitor side chains can increase this selectivity over nACE.

## Results

### Design and Synthesis

**Figure 1:**
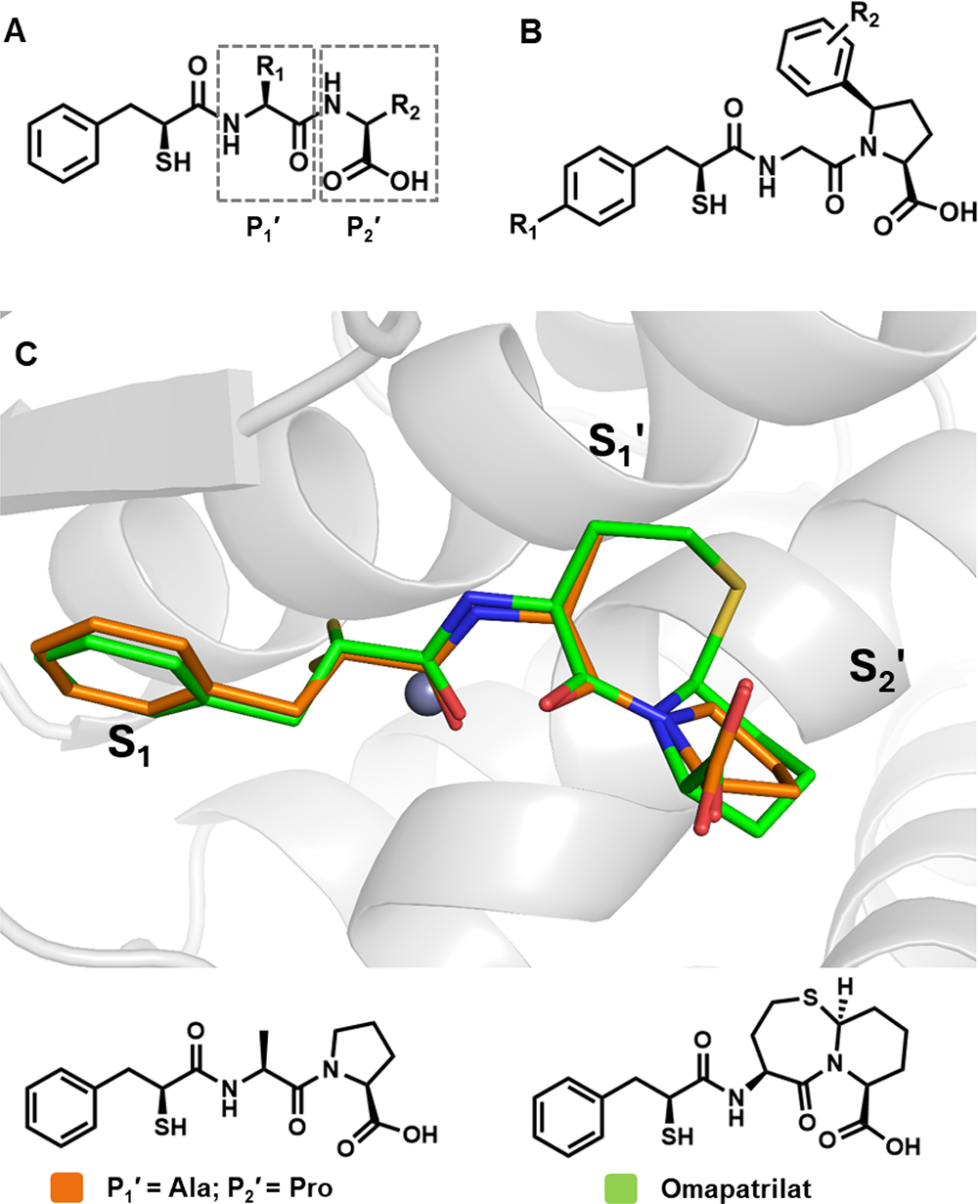
Previously reported series of dual ACE/NEP mercaptoacyl dipeptides: (A) mercapto-3-phenylpropanoyl dipeptides^23^ and (B) mercapto-3-phenylpropanoyl-glycine 5-phenylprolines.^31^ (C) Overlay of the cACE-omapatrilat (green carbon atoms) co-crystal structure (PDB code 6H5W)^32^ with a dual ACE/NEP inhibitor ((*S*)-2-methyl-3-phenylpropanoyl)-l-alanyl-l-proline (orange carbon atoms) docked into the active site of cACE. The Schechter and Berger nomenclature is used for the S_1_ to S_2_′ subsites flanking the catalytic zinc.

Coric et al. carried out SAR studies on a series of acyclic mercaptoacyl dipeptides, including 2-mercapto-3-phenylpropanoyl dipeptides (Figure 1A), which displayed potent dual ACE/NEP inhibition.^23^ Various amino acid combinations at the P_1_′ and P_2_′ positions (labeled AA_1_ and AA_2_ positions, respectively, in Coric et al.'s study) were tested for inhibitory activity against rodent testes ACE (cACE) and rabbit NEP. A number of these compounds have hydrophobic P_2_′ groups predicted to extend into the S_2_′ subsite, which may confer C-selectivity, although these compounds were not previously tested for inhibitory activity against nACE. In a related study, Fournie-Zaluski et al. carried out a SAR analysis on a different series of mercaptoacyl dipeptides made up predominantly of compounds with a 2-mercapto-3-phenylpropanoyl followed by a glycine linked to a C-terminal 5-phenylproline (Figure 1B).^31^ An overlay of the cACE-omapatrilat co-crystal structure with a dual ACE/NEP mercapto-3-phenylpropanoyl dipeptide inhibitor docked into the active site of cACE illustrates the utility of these simple peptide scaffolds for exploring the requirements of cACE/NEP inhibition (Figure 1C). In ACE, the thiol and the adjacent carboxyl form a bidentate interaction with the zinc ion, and the 3-phenylpropanoyl extends into the S_1_ subsite.

Most interactions involved in binding of peptides to NEP are found in the large hydrophobic prime subsites with the highest specificity ligands containing aromatic or bulky hydrophobic P_1_′ side chains. As a result, the binding mode of these 2-mercapto-3-phenylpropanoyl dipeptides in NEP is dependent on the residue at the P_1_′ position. In the absence of a hydrophobic side chain at this P_1_′ position, as is the case for omapatrilat, the 3-phenylpropanoyl group binds to the S_1_′ subsite, rather than the S_1_ subsite in contrast to what is observed for omapatrilat in ACE and ((*S*)-2-mercapto-3-phenylpropanoyl)-l-phenylalanyl-l-alanine in a previously reported NEP co-crystal structure.^27,32,33^

Based on these previous studies and the structures of omapatrilat and LisW, the compounds shown in Table 1 were synthesized to probe the requirements of the ACE and NEP prime subsites for cACE/NEP inhibition. This set of compounds includes 2-mercapto-3-phenylpropanoyl dipeptides AD014 and AD015 containing a flexible glycine linked to a 5-phenylproline^31^ and the mercapto-3-phenylpropanoyl dipeptide AD016 with a lysine and tryptophan in the P_1_′ and P_2_′ positions, respectively, as for LisW.

**Table 1:**
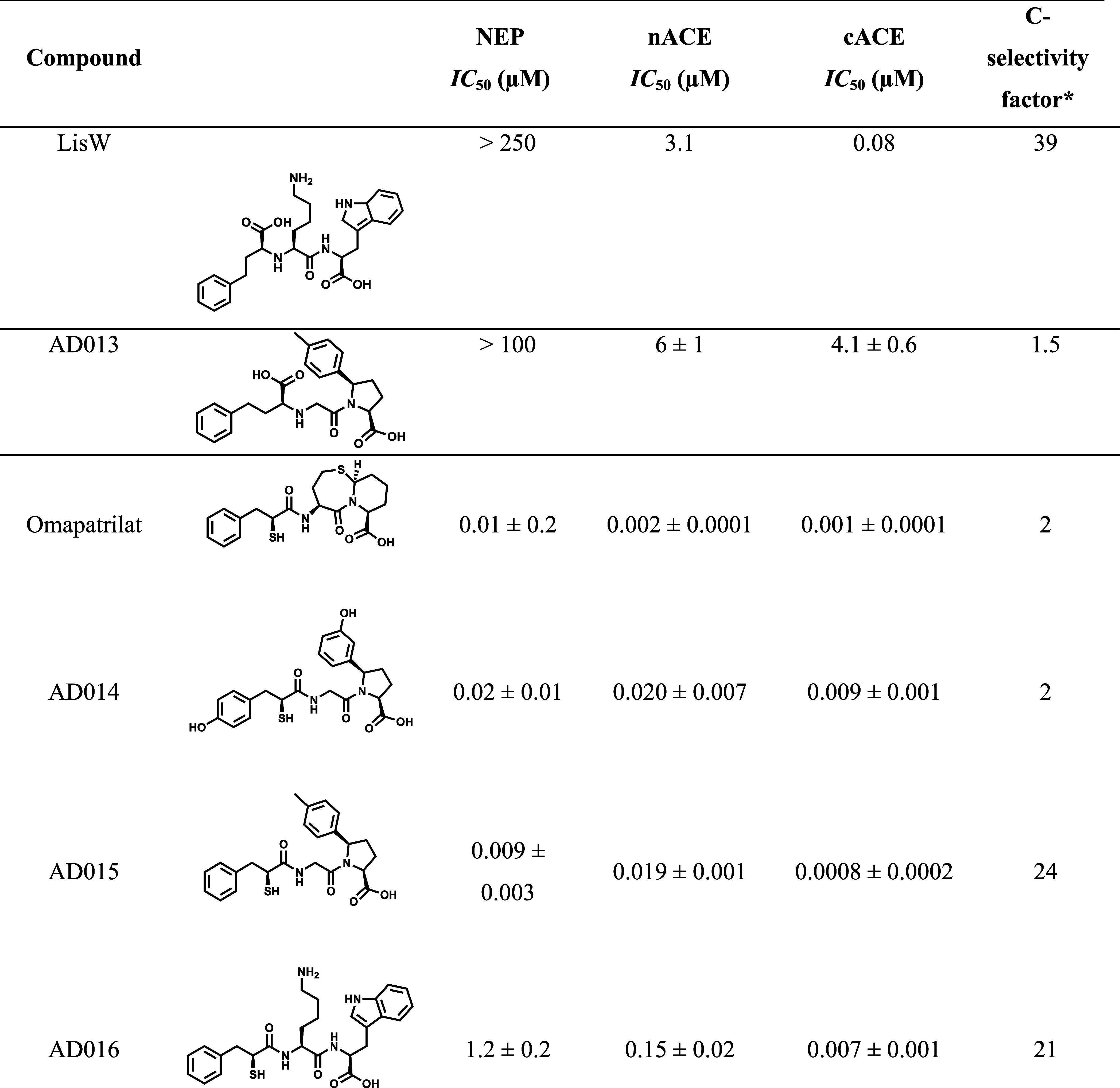
IC_50_ Values for Compounds Tested against NEP, nACE, and cACE

a IC_50_ values are reported as means ± standard errors for *n* ≥ 2 independent assays. *cACE selectivity factor = nACE IC_50_/cACE IC_50_.

**Scheme 1:**
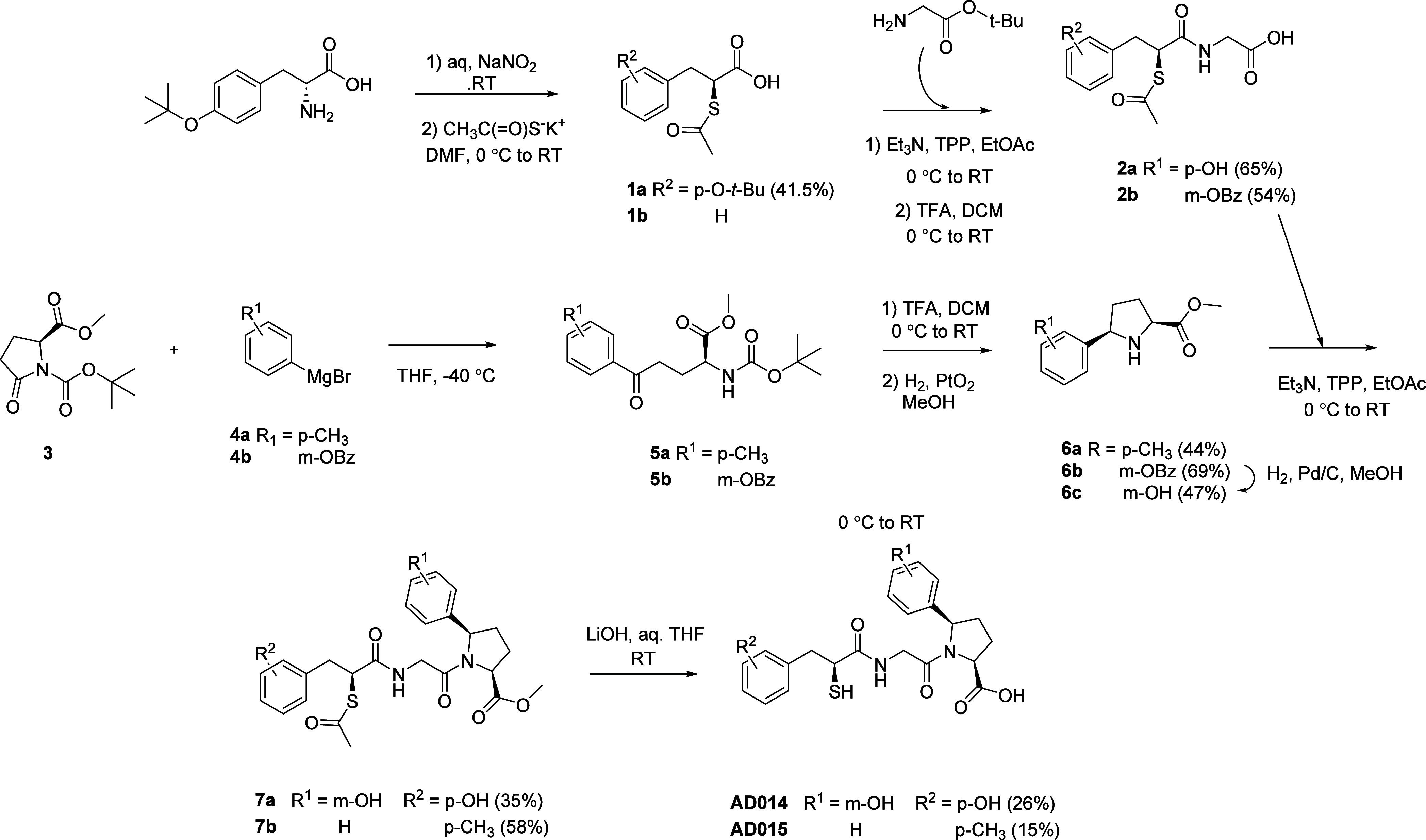
Synthesis of AD014 and AD015 Yields in parentheses.

The syntheses of AD014 and AD015 are outlined in Scheme 1. Thioacetates **1** were coupled with *t*-butylglycine followed by TFA de-esterification to afford compounds **2**. Compounds **6a** and **6c** were synthesized from **3** via Grignard addition to the valerolactam, TFA de-esterification was carried out to form the cyclic imine, and catalytic hydrogenation followed to form pyrroles **6a** and **6b**; the latter was debenzylated via catalytic hydrogenation. Peptide coupling of **2a** with **6a** and of **2b** with **6c** afforded compounds **7** that were carried on to the final products by LiOH hydrolysis.

**Scheme 2:**
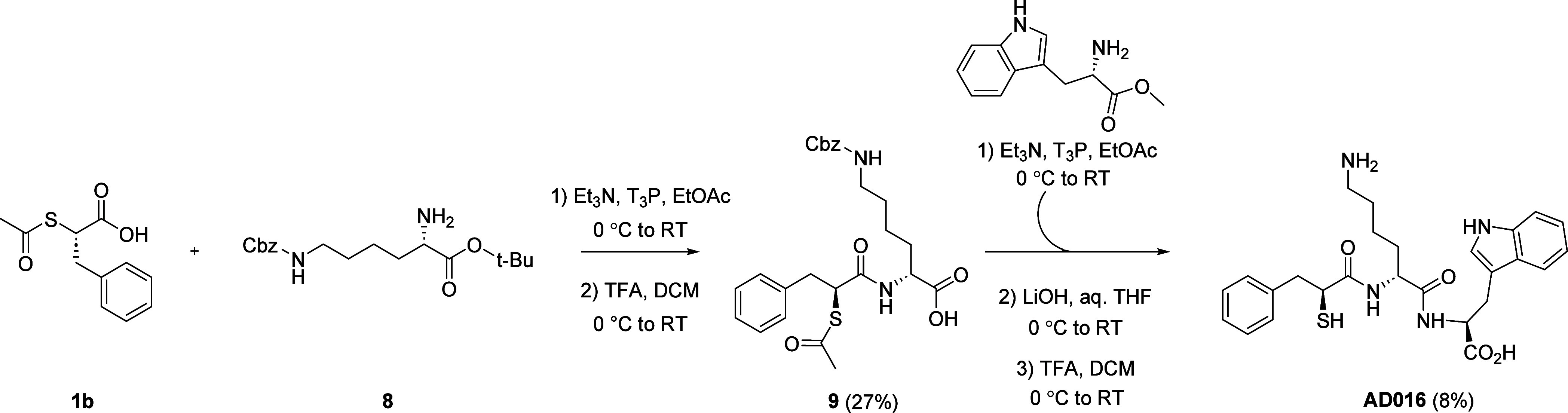
Synthesis of AD016 Yields in parentheses.

AD016 was synthesized as outlined in Scheme 2 wherein peptide coupling of acetylthiol **1b** and protected lysine **2** was followed by Boc removal to afford **3**. Subsequent peptide coupling of **3** with methyl-l-tryptophanate led to AD016 after removal of the protecting groups.

### Enzyme Inhibition

AD014, AD015, and AD016 were tested for in vitro nACE, cACE, and NEP inhibition using purified recombinant single domain human ACE proteins and the human NEP ectodomain as previously described. For an accurate comparison of the inhibitor potencies across different enzymes and substrates, it is preferable to determine inhibition constants (*K*_i_ values). Typically, for competitive inhibitors, inhibition constants can be calculated from IC_50_ values using the Cheng–Prusoff equation (*K*_i_ = IC_50_/(1 + [S]/*K*_m_)), which factors in the Michaelis–Menten constant (*K*_m_) and the substrate concentration used in the assay, provided the assay conditions conform to the classical inhibition model for competitive inhibition. Inhibition kinetics for the mercaptoacyl compounds did not conform to classical competitive inhibition models, as previously reported for omapatrilat where *K*_i_ values were determined using the Morison equation.^32^ In the case of these new analogues, it was more challenging to accurately determine *K*_i_ values that could reliably be used to determine selectivity. Detailed kinetic studies showed that compounds had slow and variable off-rates depending on the enzyme–inhibitor pair, further complicated by tight binding assay conditions in several instances (see the Supporting Information). This highlights the challenges in extrapolating data from *in vitro* assays to predict in vivo selectivity between targets. Furthermore, while comparing *K*_i_ values (assuming they can be accurately determined in vitro using available kinetic models) provides information on the relative binding affinities in the absence of substrate, in vivo the extent of enzyme inhibition will be affected by the relative concentrations and affinities of the physiological substrates competing for the binding sites. Given that accurate *K*_i_ values could not be calculated in a consistent manner for all compound-enzyme combinations, IC_50_ values are reported (Table 1). Previously reported data for LisW and AD013 and the LisW analogue with a phenylpropyl P_2_′ group^29^ are also shown in Table 1 for comparison.

### Crystal Structures of nACE and cACE in Complex with Inhibitors

High-resolution crystal structures were obtained for the inhibitors AD014 and AD015 in complex with nACE (1.85 and 1.70 Å, respectively) and cACE (1.80 and 1.80 Å, respectively) and AD016 in complex with cACE (1.45 Å). The data processing and refinement statistics for all these structures are shown in Table 2. Despite repeated efforts at multiple concentrations of the inhibitor and protein, all nACE crystals grown in the presence of AD016 did not have the ligand bound. The nACE structures crystallized in the P1 space group with two molecules in the asymmetric unit, while the cACE complex structures crystallized in the *P*2_1_2_1_2_1_ space group, with one molecule in the asymmetric unit. These are typical observations for nACE and cACE crystal structures.

**Table 2:** X-ray Data Collection and Refinement Statistics^a^

**nACE**	**AD014**	**AD015**
resolution (Å)	[74.05–10.13], (1.88–1.85)	[63.93–9.31], (1.73–1.70)
space group	*P*1	*P*1
cell dimensions (*a*, *b*, *c*)	72.93, 77.43, 82.63 Å	72.73, 76.93, 82.40 Å
cell angles (α, β, γ)	88.66, 64.29, 74.92°	88.81, 64.37, 75.32°
molecules/asymmetric unit	2	2
total/unique reflections	887,557/130,246	1,146,879/166,043
completeness (%)	[98.5], 97.5, (96.2)	[99.1], 97.3, (95.7)
*R*_merge_	[0.045], 0.105, (1.000)	[0.033], 0.082, (1.072)
*R*_pim_	[0.018], 0.043, (0.410)	[0.014], 0.033, (0.432)
⟨*I*/σ(*I*)⟩	[32.0], 10.0, (1.8)	[38.4], 10.8, (1.6)
CC_1/2_	[0.998], 0.998, (0.528)	[0.999], 0.999, (0.446)
multiplicity	[6.9], 6.8, (6.9)	[6.9], 6.9, (7.0)
Refinement statistics		
*R*_work_/*R*_free_	0.1825/0.2078	0.1799/0.2090
RMSD in bond lengths (Å)	0.006	0.009
RMSD in bond angles (°)	0.769	0.873
Ramachandran statistics (%)		
favored	98.4	98.7
allowed	1.5	1.1
outliers	0.1	0.2
Average *B*-factors (Å^2^)		
protein	39.55	39.81
ligand	58.15	58.07
water	39.10	42.32
Number of atoms		
protein	10,003	10,011
ligand	331	382
water	721	808
PDB code	9H1A	9H1B

**cACE**	**AD014**	**AD015**	**AD016**
resolution (Å)	[84.74–9.00], (1.84–1.80)	[52.34–9.00], (1.84–1.80)	[46.97–7.94], (1.47–1.45)
space group	*P*2_1_2_1_2_1_	*P*2_1_2_1_2_1_	*P*2_1_2_1_2_1_
cell dimensions (*a*, *b*, *c*)	56.75, 84.75, 134.48 Å	56.79, 85.51, 134.82 Å	56.42, 84.78, 134.11 Å
cell angles (α, β, γ)	90.00, 90.00, 90.00°	90.00, 90.00, 90.00°	90.00, 90.00, 90.00°
molecules/asymmetric unit	1	1	1
total/unique reflections	1,314,401/61,018	969,611/61,264	2,708,678/112,140
completeness (%)	[99.9], 100.0, (99.7)	[99.7], 99.4, (98.6)	[99.6], 97.8, (79.5)
*R*_merge_	[0.066], 0.286, (4.230)	[0.058], 0.258, (4.041)	[0.023], 0.97, (1.727)
*R*_pim_	[0.015], 0.063, (0.925)	[0.017], 0.066, (1.039)	[0.005], 0.020, (0.447)
⟨*I*/σ(*I*)⟩	[27.4], 10.0, (1.3)	[27.0], 9.2, (1.1)	[83.1], 22.2, (1.4)
CC_1/2_	[0.999], 0.997, (0.574)	[0.999], 0.997, (0.353)	[1.000], 1.000, (0.651)
multiplicity	[18.8], 21.5, (21.5)	[13.4], 15.8, (16.0)	[23.5], 24.2, (14.3)
Refinement statistics			
*R*_work_/*R*_free_	0.1710/0.2111	0.1667/0.2058	0.1552/0.1835
RMSD in bond lengths (Å)	0.004	0.010	0.013
RMSD in bond angles (°)	0.758	1.038	1.206
Ramachandran statistics (%)			
favored	98.3	97.9	98.8
allowed	1.5	1.9	1.0
outliers	0.2	0.2	0.2
Average *B*-factors (Å^2^)			
protein	26.29	30.04	20.76
ligand	47.57	55.23	40.59
water	33.36	35.57	33.82
Number of atoms			
protein	4823	4815	4901
ligand	154	146	130
water	502	474	676
PDB code	9H1C	9H1D	9H1E

a Inner shell, overall, and outer shell statistics are given in square brackets, unbracketed, and round brackets, respectively.

**Figure 2:**
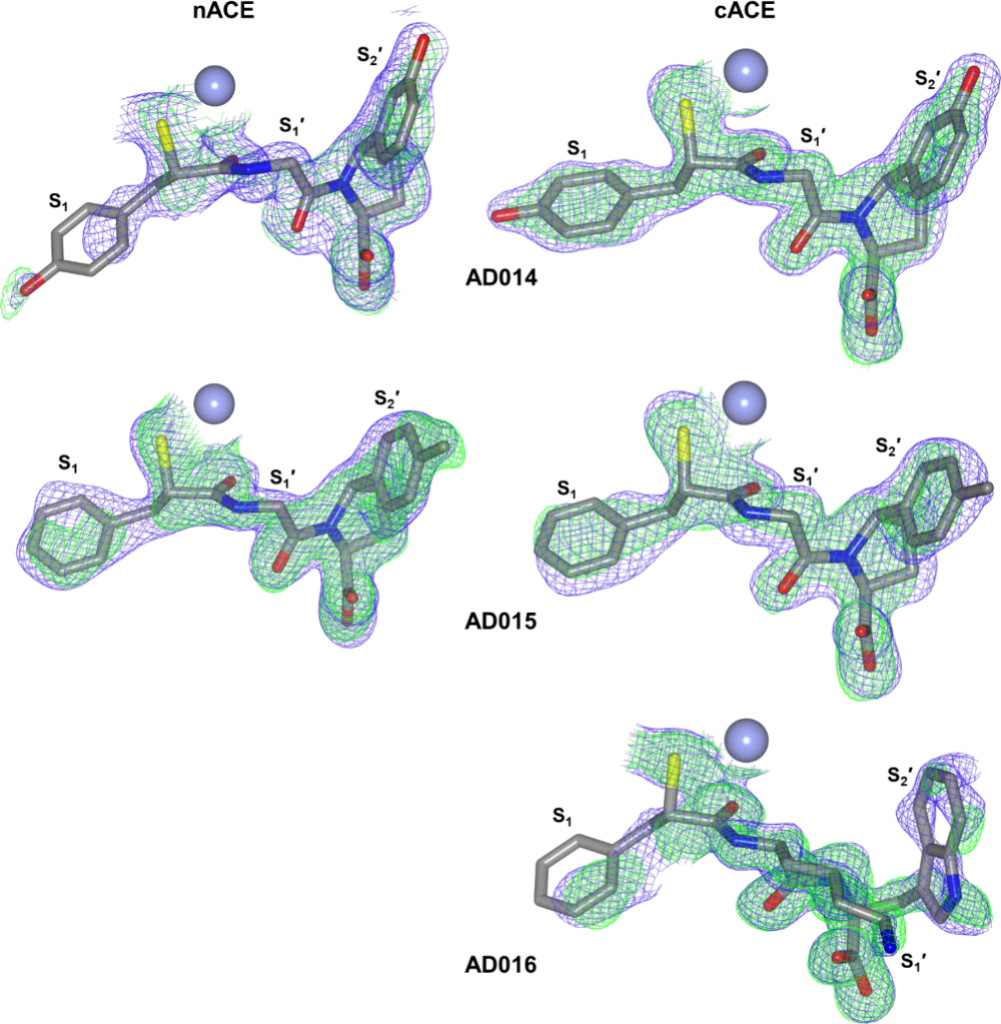
Schematic representations of AD014, AD015, and AD016 bound in the active sites of nACE and cACE. Zinc ions are shown as lilac spheres, with the final 2mFo-DFc (blue, contoured at the 1σ level) and the omit mFo-DFc (green, contoured at the 3σ level) electron density maps overlaid. The P_1_, P_1_′, and P_2_′ groups that bind in the S_1_, S_1_′, and S_2_′ subsites are indicated.

**Figure 3:**
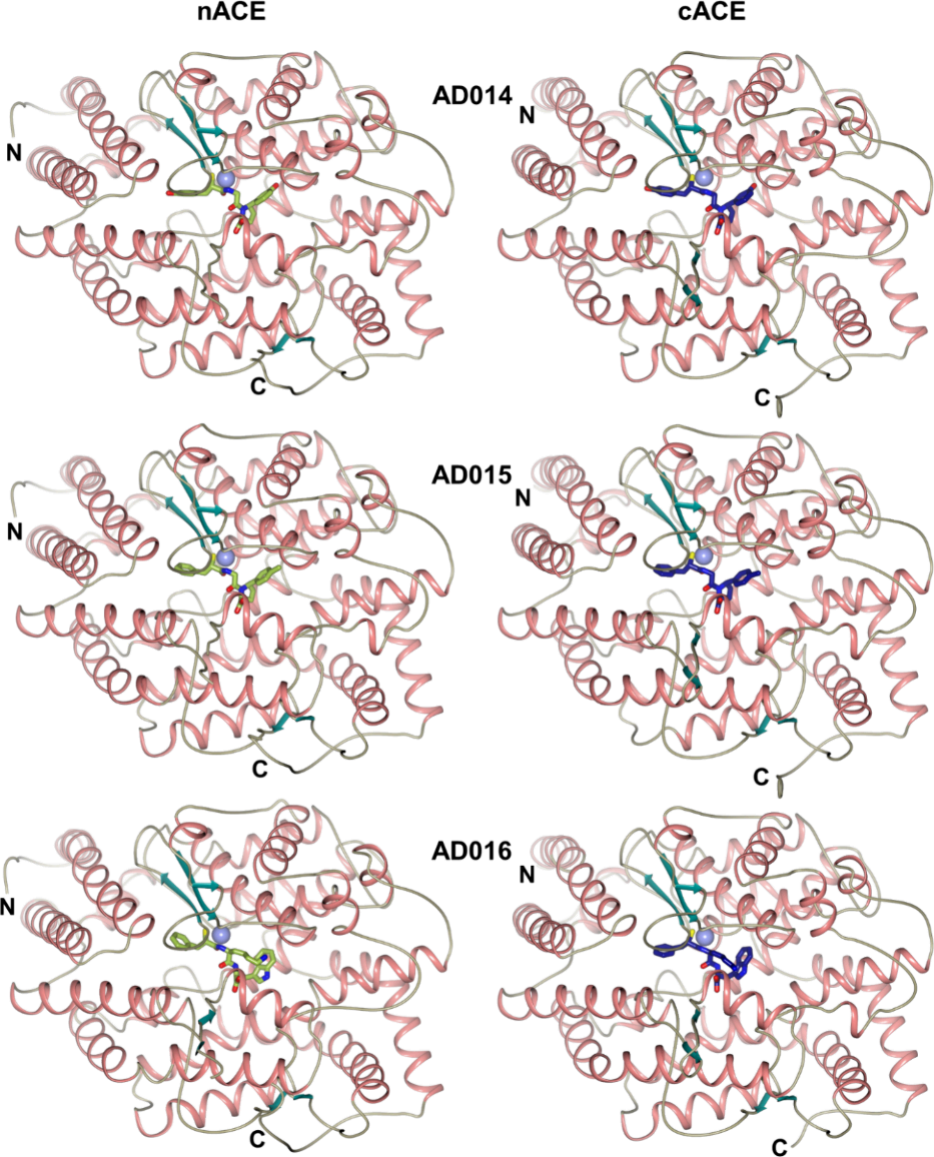
Schematic representation of the overall structures of AD014, AD015, and AD016 inhibitors in complex with nACE and cACE shown as green and blue sticks, respectively. The nACE-AD016 structure is produced by docking into nACE crystal structure PDB ID 6H5X. Zinc ions are depicted as lilac spheres, with helices, β-strands, and loops colored in rose, dark cyan, and tan, respectively.

Likewise, the overall structures of both ACE domains in all the inhibitor-bound complexes described here show the typical, mostly α-helical ellipsoid and are in the closed conformation (Figure 2). This ellipsoid is formed by two lobes that open like a clamshell to allow entry and exit from the active site that is located near the center. Access to the active site is further controlled by a flexible “lid-like” region that is made up from the first 100 residues of the ACE domains. There is very little variation observed in these main structural features between all the nACE and cACE inhibitor-bound complexes, and this is highlighted by low RMSD values observed between all structures presented here. These values range from 0.34 Å (604 Cα atoms) for the nACE structures, 0.24–0.34 Å (578 Cα atoms) for cACE structures, and a slightly higher variation of 0.90–1.00 Å (573 Cα atoms) between nACE and cACE structures (Table 3). Examination of the mFo–DFc omit maps reveals clear, unambiguous electron density for inhibitors AD014, AD015, and AD016 bound in the S_1_, S_1_′, and S_2_′ subsites of the ACE domains (Figure 3). The exception is weak density for the P_1_ groups in the nACE structures, which indicates greater flexibility due to weaker binding. These observations are reflected in the final 2mFo-DFc maps (Figure 3).

**Table 3:** Comparison of the Overall Structures of nACE and cACE in Complex with AD014, AD015, and AD016 Inhibitors^a^

		nACE		cACE		
		AD014	AD015	AD014	AD015	AD016
nACE	AD014		0.40	0.97	1.00	0.94
	AD015	0.40		0.90	0.93	0.88
cACE	AD014	0.97	0.90		0.24	0.26
	AD015	1.00	0.93	0.24		0.34
	AD016	0.94	0.88	0.26	0.34	

a RMSD values (Å) for 604, 578, and 573 Cα atoms between nACE structures, cACE structures, and nACE/cACE structures, respectively. Values were generated using the “Structure Alignment and Superposition with Gesamt” program on the CCP4 cloud.

### AD016 Binding in nACE Active Sites Predicted by In Silico Docking

Since a crystal structure of nACE in complex with AD016 was not obtained, AD016 was docked into the previously published nACE-Omapatrilat co-crystal structure (PDB 6H5X), with the resulting complex showing a similar binding pose to that observed for AD014, AD015, and AD016 in the crystal structures described above, with AD016 binding in the S_1_, S_1_′, and S_2_′ subsites (Figure 3). The inhibitor binding interactions in the active site of the crystal structures along with this docked AD016-nACE complex were examined to explain the enzyme inhibition results.

### Inhibitor Binding to the nACE and cACE Active Sites

**Figure 4:**
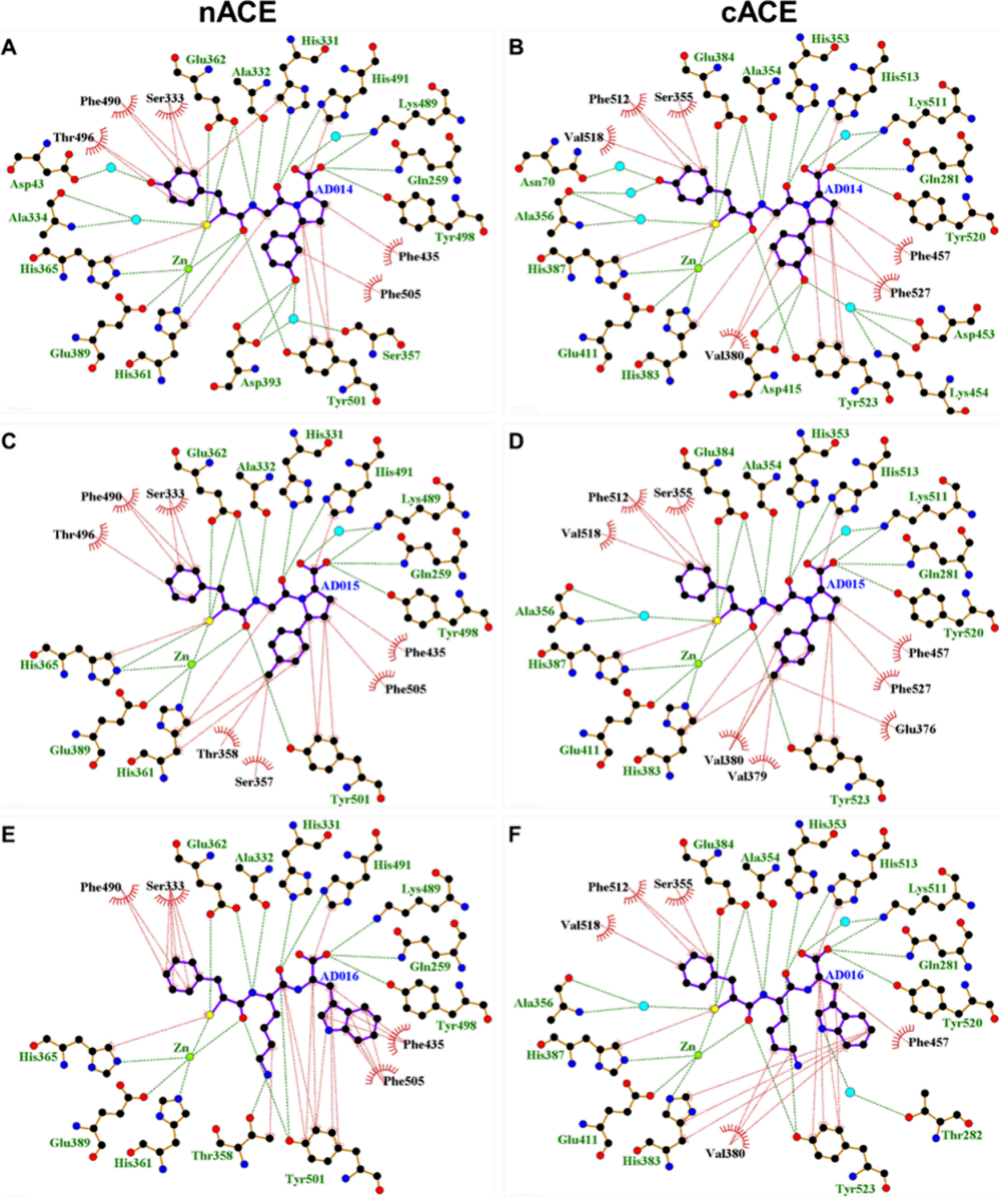
LigPlot representations of inhibitor binding interactions. Interaction comparison of (A) nACE-AD014, (B) cACE-AD014, (C) nACE-AD015, (D) cACE-AD015, and (F) cACE-AD016 complexes from crystal structures, and (E) docked nACE-AD016 complex (human nACE crystal structure PDB ID 6H5X used for docking). H-bond/electrostatic and hydrophobic interactions are shown as green and red dashed lines, respectively, and zinc ion and water molecules as green and cyan spheres, respectively. Red, semicircular symbols depict residues solely involved in hydrophobic interactions.

AD014, AD015, and AD016 share the same backbone, while the active sites of nACE and cACE show 89% identity. Therefore, it is not surprising that a comparison of the interactions of nACE and cACE with the inhibitor backbones in the active site shows that the majority are conserved for all the complexes in both the crystal and docked AD016 structures (Figure 4). The zinc ion shows a strong coordination sphere composed of the typical residues (nACE: His361, His365, and Glu389; cACE: His383, His387, and Glu411), which is completed by the P_1_ mercapto and carbonyl groups of the inhibitors. Further identically conserved interactions within all structures include the mercapto moiety of the inhibitors having a hydrogen bond with Glu362/Glu384 of nACE/cACE (this nACE/cACE nomenclature is used throughout) and a hydrophobic interaction with His365/His387. The P_1_ carbonyl also hydrogen bonds to the hydroxyl of Tyr501/Tyr523.

The inhibitors’ P_1_′ backbone amide NHs hydrogen-bond with Glu362/Glu384 and Ala332/Ala354 (backbone carbonyl oxygen atoms), while the inhibitor P_1_′ backbone carbonyl oxygen interacts with His331/His353 as well as His491/His513. The C-terminus P_2_′ carboxylate groups of the inhibitors in the crystal complexes form the typical interactions observed in ACE domain structures that are composed of direct hydrogen bonds/salt bridges with the side chains of Gln259/Gln281, Lys489/Lys511, and Tyr498/Tyr520 and a water-mediated interaction with Lys489/Lys511. The carbon atom of the P_2_′ carboxylate group also forms a hydrophobic interaction with His491/His513 in all of the complexes for all three inhibitors. In the AD016-nACE docked structure, there is a small rotation of both the P_2_′ carboxylate group and Gln259 compared to that observed in the crystal structure complexes, but the direct interaction is retained, along with those with Lys489 and Tyr498.

Apart from AD015-nACE, all other inhibitor-ACE domain crystal complexes contain a water-mediated interaction between the ligand mercapto group and the backbone amino and carbonyl groups of Ala334/Ala356. There are no significant differences between the structure of AD015-nACE and the other complexes in this region, so this lack of water-mediated interaction may be a crystallization artifact. The P_2_′ arylproline structure of AD014 and AD015 compared to the more flexible chain of AD016 results in small shifts of the prime backbone resulting in some differences in interactions. AD016 in cACE has a hydrogen bond from the P_1_′ backbone carbonyl to the Tyr523 hydroxyl and hydrophobic interactions between Tyr523 side chain and the carbon atom of the P_2_′ carboxylate group, which are also observed in the docked nACE-AD016 structure. In contrast, the AD014- and AD015-ACE domain complexes (both n- and c-) show a hydrophobic interaction between the P_1_′ backbone α-carbon and the side chain of His361/His383.

Unsurprisingly, most of the variation in interactions between the complexes are observed with the side chain binding regions of the S_1_, S_1_′, and S_2_′ subsites, and this is due to differences in both the side chains of AD014, AD015, and AD016 and domain-specific residues of nACE and cACE (Figure 4). These are described in detail in the discussion below.

### Inhibitor Binding in NEP Active Sites Predicted by In Silico Docking

**Figure 5:**
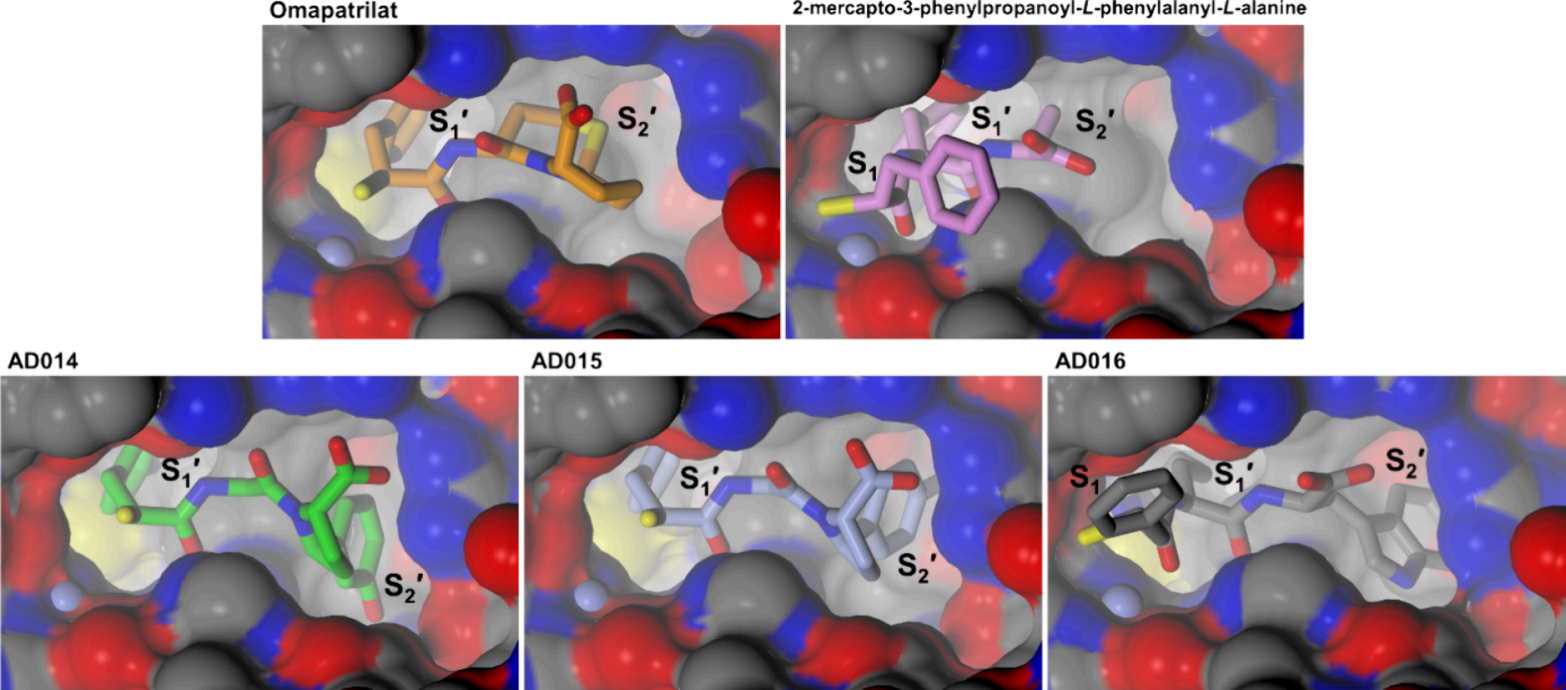
Surface representation of the active sites of NEP-((*S*)-2-mercapto-3-phenylpropanoyl)-l-phenylalanyl-l-alanine^33^ and NEP-omapatrilat (PDB code 6SUK)^27^ co-crystal structures illustrating two different binding modes in NEP. Equivalent views of the docked inhibitor-NEP complexes indicate that AD014 and AD015 bind in the same way as omapatrilat, whereas AD016 adopts an ((*S*)-2-mercapto-3-phenylpropanoyl)-l-phenylalanyl-l-alanine binding orientation. NEP crystal structure PDB ID 6SUK used for docking.

Crystallization experiments to obtain high-resolution X-ray structures of NEP in complex with AD014, AD015, and AD016 were unsuccessful. However, high-resolution structures of NEP in complex with various inhibitors, including omapatrilat, have previously been reported,^24,27,32,34,35^ so docking studies were carried out to predict the inhibitor interactions within the NEP active site using the NEP-omapatrilat co-crystal structure PDB 6SUK in both the protonated and deprotonated forms of the zinc binding thiol. The protonated and deprotonated forms of AD014 and AD015 docked with similar binding poses, with the P_1_ 3-phenylpropanoyl group extending into the S_1_′ subsite and the thiol group serving as the ZBG as observed for omapatrilat in the NEP-omapatrilat crystal structure (Figure 5). AD016 showed two different binding poses depending on the protonation state of the thiol. The deprotonated inhibitor showed the same binding orientation as omapatrilat, binding only on the prime side of the NEP active site, with the 3-phenylpropanoyl group extending into the S_1_′ subsite and the P_1_′ lysine extending into the S_2_′ subsite. In contrast, protonated AD016 adopted the same binding orientation observed with other inhibitor NEP complexes, such as ((*S*)-2-mercapto-3-phenylpropanoyl)-l-phenylalanyl-l-alanine, with the P_1_ group binding in the S_1_ pocket and the lysine side chain extending into the hydrophobic S_1_′ (Figure 5). This is also the orientation observed for AD014, AD015, and AD016 bound to nACE and cACE. Neither of the deprotonated nor protonated binding poses directly explain the poor potency of AD016 for NEP (IC_50_ = 1.2 μM), so examination of the specific interactions is required. Based on the crystal structure of the omapatrilat-NEP complex with the conserved zinc binding motif and that AD014 and AD015 contain bulky P_1_ and P_2_′ groups with a flexible P_1_′ glycine, it can be predicted that the P_1_ groups of AD014 and AD015 would likely bind in the S_1_′ pocket of NEP to maximize interactions. In contrast, AD016 contains a P_1_′ lysine group, which would need to be accommodated and is less flexible than the P_1_′ glycine residue of AD014 and AD015, so a more typical binding orientation occupying the S_1_, S_1_′, and S_2_′ subsites of NEP is likely. The docking of the protonated inhibitors into NEP showed these predicted orientations, suggesting that they are good models for explaining the relative affinities of these compounds for NEP (Figure 5) and will be the focus of the analysis. However, in vivo structural studies are required to confirm the true pose.

**Figure 6:**
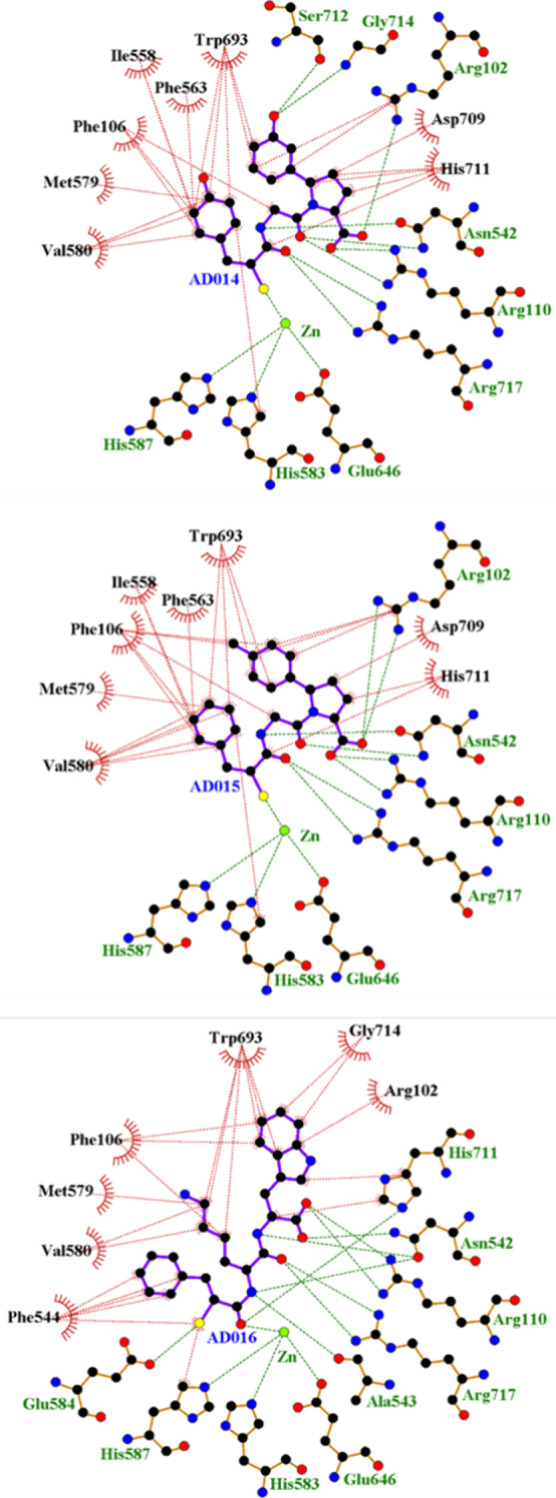
AD014, AD015, and AD016 docked into NEP. LigPlot representations of inhibitor binding interactions. H-bond/electrostatic and hydrophobic interactions are shown as green and red dashed lines, respectively, and zinc ion and water molecules as green and cyan spheres, respectively. Red, semicircular symbols depict residues solely involved in hydrophobic interactions. NEP crystal structure PDB ID 6SUK used for docking.

With AD014 and AD015 adopting the same binding orientation, they mostly share similar backbone binding interactions (Figure 6). The docking poses predict that the P_1_ mercapto group coordinates the zinc ion, while the P_1_ carbonyl group forms a bidentate hydrogen bond with Arg717 and a hydrophobic interaction with His711. Asn542 forms hydrogen bonds with both the P_1_′ backbone amide NH and carbonyl groups, and there is a hydrophobic interaction between the P_1_′ Cα and Phe106. Finally, the terminal P_2_′ carboxylate forms hydrogen bonds/salt bridges with Arg102 and Arg110. There are small differences in the positioning between the P_1_′ and P_2_′ backbone of AD014 and AD015 resulting in an additional H-bond interaction between Arg110 and P_1_′ carbonyl in AD014, compared to additional hydrogen bonds between the AD015 P_2_′ carboxylate with Arg102 and Arg110. These differences may be genuinely caused by the different P_2_′ side chains but also could be docking artifacts.

Due to AD016 binding in a different orientation to AD014 and AD015, there are differences in interactions between AD016 and NEP, although the interactions are still extensive, involving some of the same NEP residues. The P_1_ mercapto group of AD016 is predicted to form hydrophobic interactions with residues Phe544 and His587, as well as a hydrogen bond with Glu584, and the P_1_ carbonyl group interacts with the zinc ion and His711. The P_1_′ backbone amide NH can form a hydrogen bond with the Ala543 backbone carbonyl and Asn542 side chain, while the P_1_′ carbonyl has a bidendate interaction with Arg717. The P_2_′ group has a hydrophobic interaction between its Cα and His711, a bidentate interaction between its carboxylate terminus and Arg110, and Asn542 hydrogen bonds to both its backbone amide NH and its carboxylate terminus. With the backbone interactions between AD014 and AD015 being so similar and AD016 also having extensive equivalent backbone binding, the difference in affinities for NEP of these inhibitors is likely due to difference in zinc coordination (thiol for AD014 and AD015 and carbonyl for AD016) and/or variation in side chain interactions. These interactions are discussed below in relation to the affinities of the inhibitors for NEP.

## Discussion

Omapatrilat is a potent inhibitor of both ACE and NEP but does not show significant cACE selectivity over nACE. In contrast, LisW does exhibit C-selectivity in ACE but has low affinity for NEP (*K*_i_ of >150 μM). All the inhibitors presented here contain the same zinc coordination as seen with omapatrilat comprising the 2-mercapto group and P_1_ backbone carbonyl. Overall, the 2-mercapto-containing inhibitors generally displayed more potent inhibition for all three enzymes than the corresponding 2-carboxy derivatives (AD015 vs AD013 and AD016 vs LisW). Compared to omapatrilat, all the inhibitors retain very high potency for cACE, while AD014 and AD015 retain an equivalent high affinity for NEP. While all the inhibitors still retain nanomolar IC_50_ values for nACE, albeit higher than the 2 nM observed for omapatrilat, both AD015 and AD016 show moderate C-selectivity factors of 24 and 21, respectively. While AD016 showed over 200-fold improved NEP inhibitory activity (IC_50_ = 1 μM) compared to LisW (IC_50_ > 250 μM), it was not selective for NEP over nACE (1 μM compared to 0.130 μM for nACE). Compound AD015 showed a moderate 2-fold selectivity for the NEP over nACE. Closer examination of the crystal structures can provide insights into what causes these differences.

As described above, most interactions involving the backbone of the inhibitors are conserved, with variation in inhibitor side chains as well as structural differences in the nACE, cACE, and NEP S_1_, S_1_′, and S_2_′ subsites causing the range of affinities observed (Figures 4 and 6).

### Comparison of Inhibitor Binding within nACE and cACE

**Figure 7:**
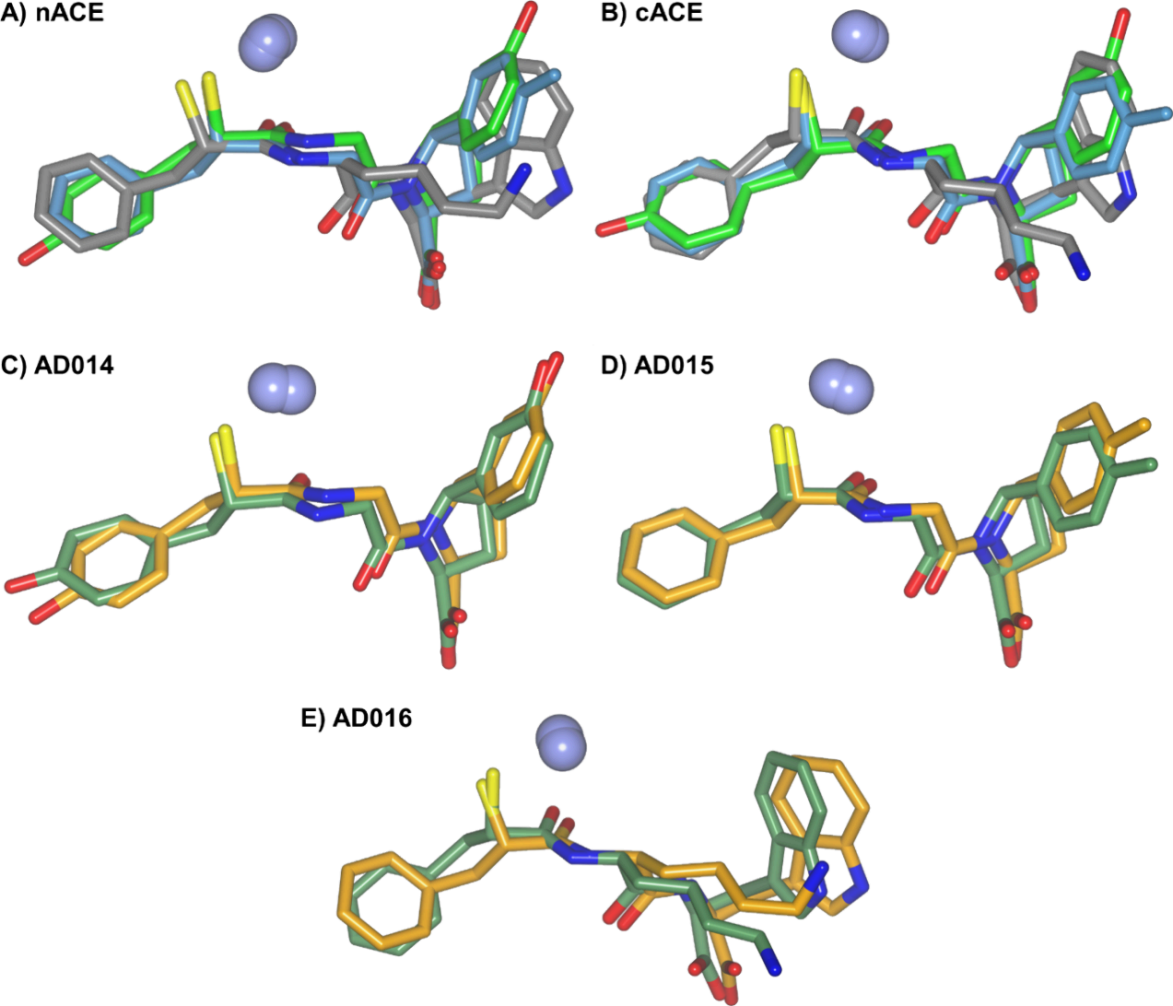
Comparison of mercapto-3-phenylpropanoyl dipeptide inhibitor binding. (A, B) Overlay of bound AD014 (green), AD015 (light blue), and AD016 (gray) inhibitors within nACE and cACE domains, respectively. (C–E) Overlay of nACE (light orange) and cACE (green) structures in complex with AD014, AD015, and AD016 inhibitors. The nACE-AD016 structure is produced by docking into the nACE crystal structure PDB ID 6H5X. Zinc ions are depicted as lilac spheres.

An overlay of AD014, AD015, and AD016 bound in nACE and cACE (AD016-nACE is a docked structure) shows similar binding positions and orientations of the inhibitors (Figure 7A,B), with the main differences being observed due to the P_1_, P_1_′, and P_2_′ side chains. AD016 bound to cACE does show small changes in the backbone orientation compared to the other two inhibitors in cACE, but again, the largest differences are observed with the side chains, particularly the addition of a P_1_′ lysine compared to glycine for AD014 and AD015. Comparison of AD014 and AD015 binding between nACE and cACE (Figure 7C,D) also shows similar binding positions and orientations. There are small differences in the P_1_ of AD014 and P_2_′ of AD015 side chains, but it will be structural differences within the nACE and cACE subsites that results in the affinity differences between nACE and cACE. While AD016 docked into nACE binds with a similar overall orientation to AD016 bound to cACE such that the inhibitor backbone overlays closely, with only a small rotation difference in the P_2_′ terminal carboxyl, there are significant differences in the P_1_, P_1_′, and P_2_′ side chain orientations (Figure 7E).

Looking at the side chain binding regions in more detail (Figure 4), all three inhibitors in the crystal structures have hydrophobic interactions between the P_1_ side chains and residues Ser333/Ser355, Phe490/Phe512, and Thr496/Val518. In addition, the side chain hydroxyl of AD014 has a water-mediated interaction with Asp43/Asp70 in both nACE and cACE and a second water-mediated hydrogen bond with the backbone carbonyl of Ala356 in cACE. The structure of AD016 docked into nACE showed a significant shift of the P_1_ phenyl group away from Thr496 toward Ser333 compared to the P_1_ groups in all the crystal structures. Due to the nACE-Thr496 to cACE-Val518 mutation, the S_1_ subsite of cACE is more hydrophobic than in nACE, which may cause the shift in the AD016 P_1_ side chain in the nACE docked structure. The increased hydrophobicity of the S_1_ subsite in cACE compared to nACE is likely to contribute to the higher affinity of these inhibitors for cACE over nACE with their largely hydrophobic aromatic P_1_ groups. The less hydrophobic environment in nACE is also consistent with there being weak density in the 2mFo-DFc maps for the P_1_ groups of AD014 and AD015 indicating more flexible binding.

The P_1_′ residue of AD014 and AD015 is glycine; therefore, only AD016 shows interactions in the S_1_′ subsite. These consist of two hydrophobic interactions from the lysine side chain of AD016 with Val380 of cACE. This residue is Thr358 in nACE, which forms a less hydrophobic environment for the carbons of the P_1_′ lysine side chain. This may contribute to not only the lower affinity of AD016 for nACE compared to cACE but also AD016 having a lower affinity for nACE than both AD014 and AD015. The less hydrophobic Thr358 in nACE causes the lysine side chain of AD016 to have a conformation different from that observed in the AD016-cACE crystal structure (Figure 7E) such that the amino group bends back on itself to form a hydrogen bond with the Thr358 side chain hydroxyl. This variation in the AD016 P_1_′ side chain orientation between nACE and cACE also causes a difference in the P_2_′ side chain orientation between the AD016-nACE docked structure and AD016-cACE crystal structure.

**Figure 8:**
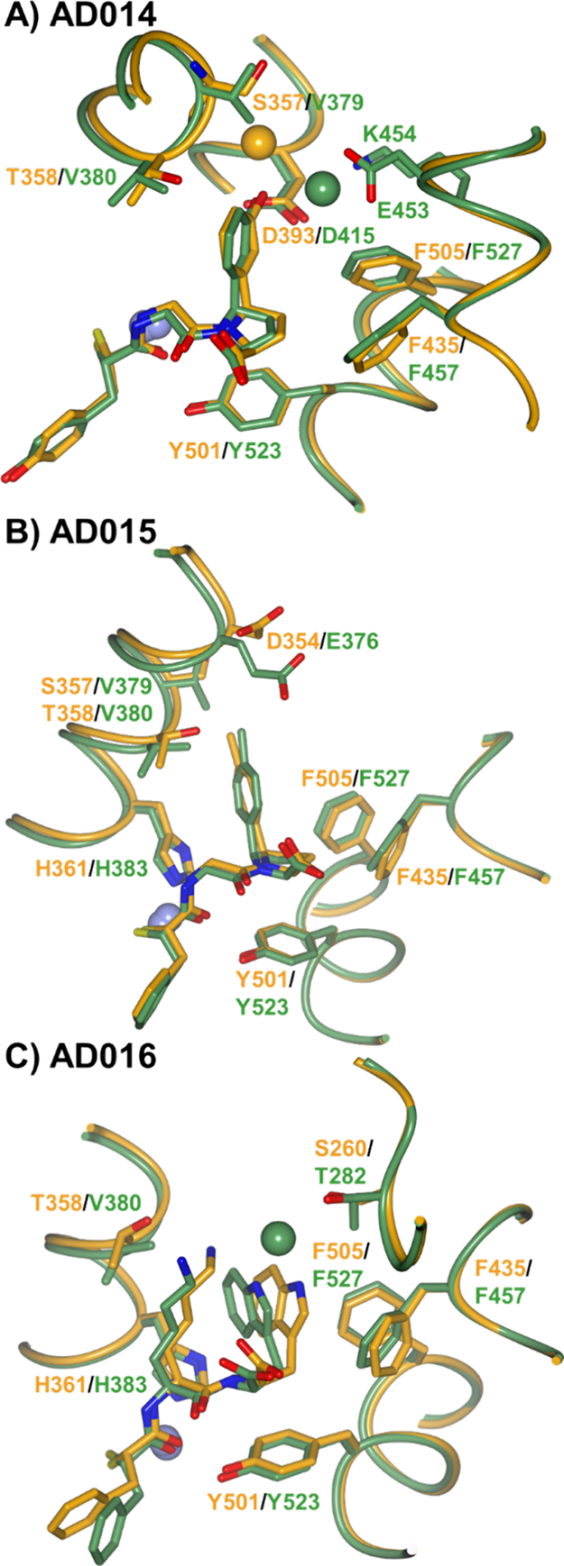
Residue differences between nACE and cACE in the S_2_′ subsite affect inhibitor affinity. Overlay of nACE (light orange) and cACE (green) structures in complex with (A) AD014, (B) AD015, and (C) AD016 inhibitors. The nACE-AD016 structure is produced by docking into nACE crystal structure PDB ID 6H5X. Zinc ions are depicted as lilac spheres, with water molecule spheres colored light orange (nACE) and green (cACE).

The greatest variation in interactions between the ACE domains and the different inhibitors occurs in the S_2_′ subsite (Figures 4, 7, and 8). In all the inhibitor-ACE domain complex structures, there are hydrophobic interactions between the P_2_′ Cβ atom and Phe435/Phe457, while AD014 and AD015 also have a further hydrophobic interaction (two interactions in the AD014-cACE structure) between the P_2_′ side chain and Phe505/Phe527 in both ACE domains. The P_2_′ side chain conformation in the AD016-nACE docked structure is orientated toward Phe505 to form 3.8 Å edge to face hydrophobic interaction, while in the cACE crystal structure, the P_2_′ side chain has a hydrophobic environment from Phe527, but the orientation is not ideal for edge to face interaction and the distance is greater at 4.5 Å. All complexes contain hydrophobic interactions between the inhibitor side chains and Tyr501/Tyr523, with AD016 also interacting via its backbone carbon atom and Tyr501/Tyr523. While AD014-nACE has no further hydrophobic interaction in the S_2_′ subsite, it has a bidentate interaction from the P_2_′ hydroxyl with Asp393 and water-mediated hydrogen bonds with Ser357 and Asp393 (Figures 4 and 8A). In contrast, in cACE, there are additional hydrophobic interactions between the P_2_′ aromatic ring and Val380, the equivalent bidentate interaction with Asp415, and alternative water-mediated hydrogen bonds with Asp453 and Lys454. Therefore, although AD014 binding benefits from the more hydrophobic environments around the P_2_′ aromatic ring in cACE (Val380 compared to Thr358 in nACE), as well as the previously mentioned more hydrophobic S_1_ subsite, the affinity for nACE is equivalent. A possible explanation for this is the presence of Val379 in cACE positioned close to the P_2_′ hydroxyl of AD014, this being a bulkier and more hydrophobic residue than the equivalent Ser357 of nACE.

In the cACE complex, the P_2_′ *p*-tolyl group of AD015 benefits from being in a hydrophobic environment resulting in extensive interactions with the acyl chain of Glu376, Val379, Val380, and His383 (Figures 4 and 8B). The fully hydrophobic nature of the P_2_′ *p*-tolyl group of AD015 may explain the higher affinity of AD015 for cACE compared to AD014 due to proximity between Val379 and the P_2_′ hydroxyl of AD014 described above. While the *p*-tolyl group of AD015 also shows hydrophobic interactions with equivalent residues Ser357, Thr358, and His361 in nACE, the P_2_′ environment is less hydrophobic, particularly due to Thr358. This, together with the less hydrophobic S_1_ subsite in nACE is consistent with the C-selectivity factor of 24.

The indole P_2_′ group of AD016 forms hydrophobic interactions with Val380 and His383 of cACE, and there is an additional water-mediated hydrogen bond bridge to Thr282 (Figures 4 and 8C). Although the crystal structure of nACE in complex with AD016 was not obtained, the docking model predicted that the water-mediated hydrogen bond bridge could be retained with equivalent Ser260. However, the mutation of cACE-Val380 to nACE-Thr358 results in a less hydrophobic environment. As mentioned above, the docking of AD016 into nACE showed the interaction of the P_1_′ lysine side chain with Thr358 hydroxyl, and this moved the P_2_′ tryptophan side chain away from Thr358 preventing any hydrophobic interactions. The reduction in hydrophobicity of nACE-Thr358 compared to cACE Val380 affects the binding in both the S_1_′ and S_2_′ subsites, in addition to the less hydrophobic S_1_ subsite, explains the C-selectivity factor of 21.

### Comparison of Inhibitor Binding in NEP

Examination of the interactions of AD014, AD015, and AD016 in the NEP active site can help explain the different binding affinities (Table 1 and Figure 6). The S_1_ subsite of NEP is open and not well-defined, resulting in fewer interactions than observed in the prime subsites. AD016 is the only inhibitor predicted by docking to have its P_1_ side chain located in the S_1_ subsite, where it has hydrophobic interactions with Phe-544. While AD014 and AD015 lack these interactions, their P_1_ side chains are bound deep within the hydrophobic S_1_′ subsite. AD015 has a network of hydrophobic interactions between its P_1_ group and residues Phe106, Ile558, Phe563, Met579, Val580, and Trp693. AD014 retains these interactions, but the hydroxyl located in this hydrophobic pocket likely contributes to the lower affinity of AD014 for NEP relative to AD015. In the predicted binding orientation of AD016 in NEP, the thiol-zinc interaction is replaced by a weaker carbonyl zinc interaction, and the P_1_′ lysine group binds in the hydrophobic S_1_′ subsite. While the P_1_′ lysine carbons are predicted to form hydrophobic interactions with Phe106, Phe544, Met579, Val580, and Trp693, these are not only less than observed with AD014 and AD015, but the highly hydrophilic lysine amino group is even less suited to being bound in the hydrophobic environment. These observations are consistent with AD016 having the lowest affinity for NEP.

The P_1_′ glycine of AD014 and AD015 does not bind in any subsite but does form a hydrophobic interaction with Phe106. There are further differences between the inhibitors in the binding of the P_2_′ groups within the S_2_′ pocket. The AD014 P_2_′ group forms a network of hydrophobic interactions with Arg102, Trp693, Asp709, and His711, as well as two hydrogen bonds from its carbonyl group with Ser712 and Gly714 from its carbonyl group. In comparison, AD015 retains similar hydrophobic interactions, but instead of hydrogen bonds, it has extra hydrophobic interactions with Phe106. The P_2_′ tryptophan group of AD016 is well-suited to maximize interactions in the S_2_′ pocket, where it has hydrophobic interactions with Arg102, Phe106, Trp693, His711, and Gly714. There is the potential of water-mediated interactions between the P_2_′ tryptophan and Tyr697, Asp709, and Ser712 backbone carbonyl. From the data available, it is difficult to judge whether AD014, AD015, or AD016 has the stronger binding in the S_2_′ pocket, but it suggests that the largest effect on affinity results from the S_1_′ subsite.

### Examination of Structural Features Leading toward Dual cACE/NEP Inhibition

**Figure 9:**
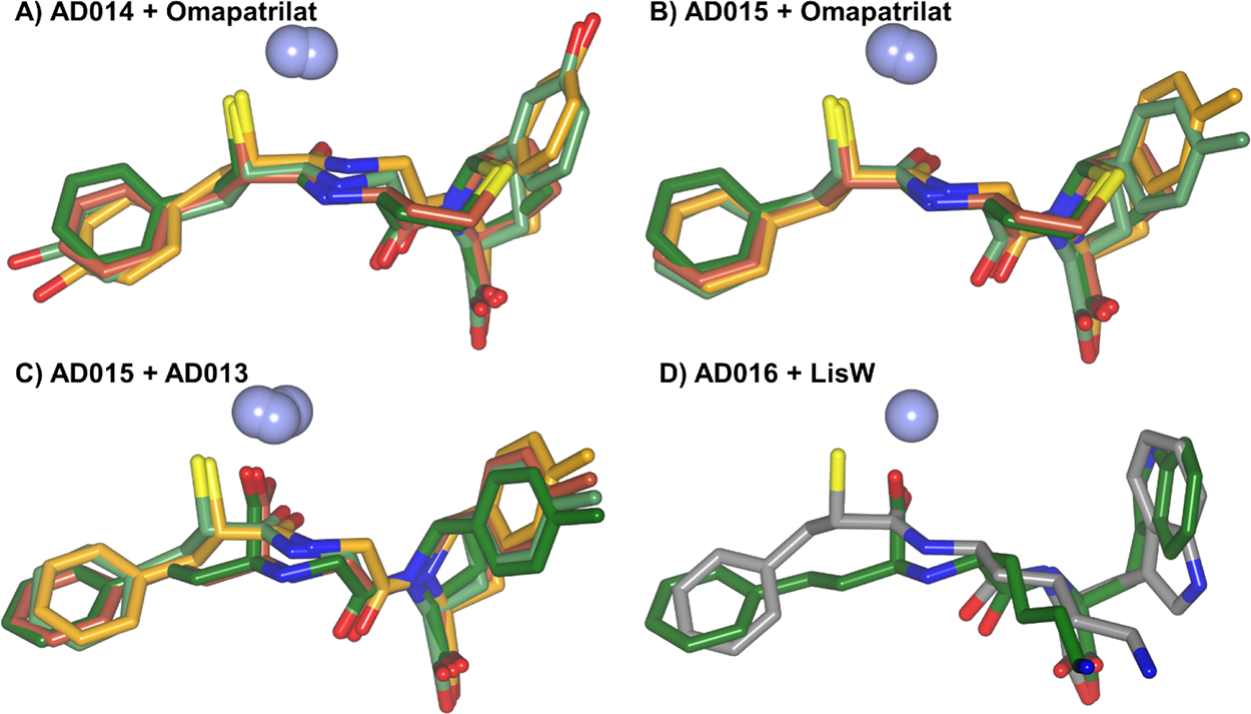
Comparison of mercapto-3-phenylpropanoyl dipeptide inhibitors binding with omapatrilat and equivalent carboxy-3-phenylpropyl dipeptides. Overlay of nACE (orange) and cACE (green) structures in complex with (A) AD014 (light) + omapatrilat (dark), (B) AD015 (light) + omapatrilat (dark), (C) AD015 (light) + AD013 (dark), and (D) (only in cACE) AD016 (green) + LisW (gray) inhibitors. Zinc ions are depicted as lilac spheres.

Combining this detailed analysis of affinity and binding of AD014, AD015, and AD016 to nACE, cACE, and NEP with comparison to previous inhibitors can indicate what structural features are important for a selective cACE/NEP inhibitor. First, an overlay of AD014 and AD015 with omapatrilat (Figure 9A,B) shows similar binding orientations in all three enzymes, indicating that any affinity differences are likely caused by the changes in inhibitor side chains. AD014 has an additional hydroxyl group on the P_1_ side chain compared to omapatrilat, as well as another hydroxyl on the P_2_′ group with omapatrilat having a fused ring P_1_′/P_2_′ system. From the data, it is unclear whether it is one or both changes that cause the ∼10× reduction in affinity of AD014 for both nACE and cACE compared to omapatrilat, and it also does not improve the cACE selectivity. This combination of changes also has a small detrimental effect on NEP affinity, but considering that the more hydrophilic P_1_ phenol of AD014 likely binds less strongly in the S_1_′ subsite than the P_1_ phenyl of omapatrilat, it does suggest that the P_1_′ glycine and P_2_′ side chain of AD014 may improve NEP binding relative to the fused ring P_1_′/P_2_′ system of omapatrilat. AD015 has a conserved P_1_ group with omapatrilat; therefore, the only change is the P_1_′/P_2_′ region. The affinity for NEP is nearly equivalent; therefore, it is interesting to speculate whether an inhibitor in which the P_1_ hydroxyl of AD014 is removed would have a higher affinity for NEP than omapatrilat. AD015 has an equivalent affinity for cACE as observed for omapatrilat, but the affinity for nACE is reduced by a factor of 10. This is likely largely due to nACE not accommodating large hydrophobic P_2_′ side chains.

We compared the AD014, AD015, and AD016 structures presented here with those of LisW and three LisW analogues that we previously reported.^29^ This analysis provided two interesting comparisons between AD015 and the LisW analogue AD013 (Figure 9C), as well as between AD016 and LisW (Figure 9D). These both have conserved P_1_, P_1_′, and P_2_′ groups within each comparison and only differ in the ZBG. Both AD015 and AD016 have a thiol and carbonyl ZBG compared to the P_1_ carboxylate group of AD013 and LisW. These comparisons show that inhibitors containing thiol-carbonyl zinc coordination bind significantly more strongly to nACE, cACE, and in particular NEP than the equivalent P_1_ carboxylate-containing compounds. The overlays show that the inhibitor backbones of AD013 and LisW are positioned further from the zinc ion than the backbone atoms in AD015 and AD016, and this also causes variation in the side chain orientation and position. Therefore, it could be a combination of zinc binding strength and optimal side chain orientation and position that is important in developing high affinity inhibitors.

## Conclusions

In summary, high-resolution crystal structures, in silico docking, and enzyme inhibition data from this study provide important insight into the structural requirements for dual cACE/NEP inhibition. Two moderately C-selective ACE inhibitors have been identified, and while they are not sufficiently selective for NEP over the nACE, they do show greatly improved NEP potency compared to LisW and AD013. These inhibitors confirm that to increase NEP affinity, a hydrophobic group binding in the S_1_′ subsite is important. This can be achieved by two approaches, first by having a hydrophobic P_1_′ group or alternatively having a P_1_′ glycine, which along with a mercapto-3-phenylpropanoyl backbone allows a hydrophobic P_1_ side chain to bind in the S_1_′ subsite of NEP. Having a hydrophobic P_1_′ side chain not only increases NEP affinity but also provides some C-selectivity due to proximity to the Thr358/Val380 mutation in the S_1_′ subsite of nACE and cACE. This can be combined with the bulky hydrophobic P_2_′ side chain to further increase cACE selectivity. Without extending further into the nonprime binding subsites, which would make a potential inhibitor large and less drug-like, it has been difficult to find an inhibitor P_1_ side chain that confers any cACE selectivity. Therefore, the P_1_ group can likely only be used to increase overall affinity.

While the mercapto-3-phenylpropanoyl backbone is significantly better for NEP potency than a P_1_ carboxylate-containing inhibitor and improves cACE affinity, it may be more difficult to dial out nACE binding due to the tight binding nature of the thiol-carbonyl ZBG in ACE. Further investigation is needed to identify other ZBGs that provide a binding affinity between what is observed for thiol-carbonyl and P_1_ carboxylate or allosteric inhibitors that do not interact with the zinc. This would have a better prospect of retaining high affinity for cACE and NEP but allows for greater effects from P_1_′ and P_2_′ side chains to reduce affinity for nACE.

## Experimental Section

### Chemistry

Commercial reactants and reagents were acquired from Enamine, Combi-Blocks, Sigma-Aldrich, or Fluorochem without further purified. Reaction progress was monitored by TLC or LC/MS (liquid chromatography/mass spectroscopy). Flash chromatography purifications were carried out on a Biotage Isolera Flash Chromatography instrument using SiO_2_ 60 (230–400 mesh, particle size 0.040–0.055 mm) columns. Final derivatives for biological testing were purified to ≥95% as ascertained on a 1260 HPLC/Agilent 1200 MS using an XBridge C6 column, a 95:5 mobile phase gradient over 6 min of 0.1% trifluoroacetic acid in acetonitrile, and 0.1% trifluoracetic acid in water at a flow rate of 1.5 mL/min. Peaks were analyzed via a detector diode array (DAD), and an Agilent 6120 quadrupole (single) mass spectrometer operating in both APCI and ESI ionization modes. ^13^C and ^1^H NMR spectra were determined at 75.5 and 400 MHz, respectively, on a Bruker spectrometer at ambient temperature (unless stated otherwise). Chemical shifts (δ) are reported in ppm downfield from the internal standard tetramethylsilane. Coupling constants, *J*, are reported in hertz (Hz).

#### Synthesis of (2*S*,5*R*)-5-(3-Hydroxyphenyl)-1-(((*S*)-3-(4-hydroxyphenyl)-2-mercaptopropanoyl)glycyl)pyrrolidine-2-carboxylic Acid (AD014)

##### Step 1: Methyl (*S*)-5-(3-(Benzyloxy)phenyl)-2-((*tert*-butoxycarbonyl)amino)-5-oxopentanoate (**5b**)

A catalytic amount of I_2_ (0.417 g, 1.64 mmol) was added to 1-(benzyloxy)-3-bromobenzene (5.19 g, 0.0197 mol) and magnesium (0.8 g, 0.0329 mol) in THF (50). The mixture was stirred at ambient temperature for 1 h before cooling to −40 °C and adding a 10 mL of THF solution of Boc-l-pyroglutamic acid methyl ester (4 g, 0.0164 mol). The mixture was stirred with slow warming to room temperature over 6 h. After quenching with saturated aq. NH_4_Cl solution, the mixture was extracted twice with 100 mL of EtOAc. The combined organic layers were dried (Na_2_SO_4_) and concentrated. Purification by flash chromatography (eluting with 30% ethyl acetate in hexane) afforded **5b** as a brownish solid. Yield: 6 g, 85%. LCMS (M+H)^+^: 328.2 (fragment due to loss of *t*-butoxycarbonyl).

##### Step 2: Methyl (*S*)-5-(3-(Benzyloxy)phenyl)-3,4-dihydro-2*H*-pyrrole-2-carboxylate

**5b** (6 g, 0.014 mol) in DCM (60 mL) and 10.7 mL of trifluoroacetic acid were combined at 0 °C and warmed to rt with stirring for 16h. After removal of solvent, the residue was dissolved in DCM, which was then removed in vacuo to give the title compound as a yellowish liquid. The residue was used in the next step without purifying further. Yield: 3.2 g; 74%.

##### Step 3: Methyl (2*S*,*5R*)-5-(3-(Benzyloxy)phenyl)pyrrolidine-2-carboxylate (**6b**)

A mixture of the preceding compound (3.2 g, 0.0103 mol) and PtO_2_ (0.32 g, 10% w/w) in MeOH (30 mL) was hydrogenated under H_2_ (pressure 140 psi) over 4 h. The reaction mixture was passed through Celite rinsing through with EtOAc. Removal of solvent from the filtrate afforded **6b** as a yellowish gummy solid that was used subsequently without further purification. Yield: 3 g; 93%.

##### Step 4: Methyl (2*S*,5*R*)-5-(3-Hydroxyphenyl)pyrrolidine-2-carboxylate (**6c**)

A solution of **6b** (3 g, 0.0096 mol) and Pd–C (10%) (0.3 g, 10% w/w) in MeOH (30 mL) was hydrogenated under H_2_ (pressure 140 psi) over 6 h. The mixture was filtered through Celite rinsing with EtOAc. After removal of solvent, the residue was purified by flash chromatography using EtOAc and pet ether as eluents to give **6c** as a yellowish gummy solid. Yield: 1 g; 47%. LCMS (M+H)^+^: 222.2; ^1^H NMR (*d*_6_-DMSO): δ 9.2 (s, 1H), 7.2 (t, *J* = 8 Hz, 1H), 6.8 (d, *J* = 8 Hz, 1H), 6.06 (dd, *J* = 8, 2 Hz, 1H), 4.0–4.1 (m, 1H), 3.8 (m, 1H), 3.7 (s, 1H), 3.0 (br s, 1H), 2.0–2.1(m, 2H), 1.9–2.0 (m, 1H), 1.6 (m, 1H).

##### Step 5: (*R*)-2-Bromo-3-(4-(*tert*-butoxy)phenyl)propanoic Acid

A solution of NaNO_2_ (2.2 g, 0.031 mol) in water was added dropwise to a solution of (*R*)-2-amino-3-(4-(*tert*-butoxy)phenyl)propanoic acid (4 g, 0.016 mol) in 48% aq. HBr (6 g, 0.074 mol) cooled to −20 °C. Then, an aq. solution of KBr (7.43 g, 0.062 mol) was added dropwise, and the mixture was stirred for 30 min. Solids that precipitated were filtered, washed with hexane, and dried under high vacuum to provide the title compound as a light brown solid. The solid was used subsequently without purifying further. Yield: 2.5 g; 50%. LCMS (M–H)^−^: 299.3; 301.3.

##### Step 6: (*S*)-2-(Acetylthio)-3-(4-(*tert*-butoxy)phenyl)propanoic Acid (**1a**)

Potassium thioacetate (5.6 g, 0.049 mol) was added to an ice-cooled solution of the preceding compound (5 g, 0.016 mol) in DMF (15 mL), and the mixture was warmed to rt over 1 h. The reaction mixture was quenched with water and extracted several times with EtOAc. The organic layers were dried (Na_2_SO_4_) and concentrated affording **1a** as a light brown solid, which was used in the next step without additional purification. Yield: 4.1 g; 83%. LCMS (M–H)^−^: 295.3.

##### Step 7: *tert*-Butyl (*S*)-(2-(Acetylthio)-3-(4-(*tert*-butoxy)phenyl)propanoyl)glycinate

T_3_P in 50% EtOAc (0.84 g, 2.66 mmol) was added to **1a** (1.0 g, 3.0 mmol), TEA (3.7 mL, 36 mmol), and *tert*-butyl glycinate (1.25 g, 9 mmol) in THF (10 mL) at 0 °C. After stirring for 30 min while warming to rt, the reaction mixture was quenched with water and extracted several times with EtOAc. The combined organic layers were dried over Na_2_SO_4_, and the mixture was concentrated. The resultant solid was collected by rinsing with hexanes through a filter and dried in vacuo to give the title compound as a light brown solid. The solid was used for the next step, without additional purification. Yield: 1.2 g; 92%. LCMS (M+H)^+^: 354.1 (fragment due to the loss of isobutylene).

##### Step 8: (*S*)-(2-(Acetylthio)-3-(4-hydroxyphenyl)propanoyl)glycine (**2a**)

The preceding compound (2 g, 4.0 mmol) dissolved in ice-cooled TFA (10 mL) was stirred with warming to rt over 15 min. TFA was removed via rotary evaporation to give **2a** as a light brown solid that was propagated in the next step without purifying. Yield: 1 g; 71%. LCMS (M+H)^+^: 298.4.

##### Step 9: Methyl-(2*S*,5*R*)-1-(((*S*)-2-(acetylthio)-3-(4-hydroxyphenyl)propanoyl)glycyl)-5-(3-hydroxyphenyl)pyrrolidine-2-carboxylate (**7a**)

TEA (3.5 mL, 2.0 mmol) and 50% T_3_P in EtOAc (6 mL, 1.0 mol) were added in sequence to an ice-cooled THF (10 mL) solution of **2a** (1 g, 3.0 mmol). After the mixture was stirred at 0 °C for 30 min, methyl (2*S*,5*R*)-5-(3-hydroxyphenyl)pyrrolidine-2-carboxylate (0.996 g, 6.0 mmol, from Step 4) was added at 0 °C, which was followed by warming to rt over 30 min. Water was added to the reaction mixture, and solids that precipitated were filtered, washed with hexane, and dried in vacuo affording **7a** as a light brown solid. The product was used subsequently without further purification. Yield: 0.69 g; 35%.

##### Step 10: (2*S*,5*R*)-5-(3-Hydroxyphenyl)-1-(((*S*)-3-(4-hydroxyphenyl)-2-mercaptopropanoyl)glycyl)pyrrolidine-2-carboxylic Acid (AD014)

LiOH (0.29 g, 0.0069 mol) was added to a solution of **7a** (0.69 g, 1.3 mmol) in THF (5 mL) and water (5 mL), and the resultant mixture was stirred at rt for 2h. The mixture was taken up in water with extraction several times by EtOAc. The organic layers were dried (Na_2_SO_4_) and concentrated. The residue was purified by flash chromatography (eluting with 5–7% methanol in chloroform), followed by preparatory HPLC to afford AD014 as an off-white solid. Yield: 160 mg; 26%. LCMS (M+H)^+^: 445.1.

^1^H NMR (*d*_6_-DMSO, 356 °K): δ 7.8 (m 1H), 6.9–7.2 (m, 6H), 6.65 (m 4H), 5.0 (m, 1H), 4.4–4.5 (m, 1H), 3.8–4.0 (m, 1H), 3.15–3.3 (m, 1H), 3.05 (dd, *J* = 14, 7.5 Hz, 1H) 0–3.2 (m, 1H), 2.3–2.45 (m, 2H), 2.6–2.7 (m, 1H), 2.3–2.5 (m, 1H), 2.15–2.25 (m, 1H), 1.9–2.0 (m, H), 1.8–1.9 (m, H) ^13^C NMR (*d*_6_-DMSO): δ 171.5, 172.4, 168.3, 157.7, 156.0, 144.8, 130.4, 130.1, 129.2, 117.3, 115.3, 114.6, 113.5, 61.7, 60.8, 42.8, 41.8, 40.8, 36.1, 27.4.

#### Synthesis of (2*S*,5*R*)-1-(((*S*)-2-Mercapto-3-phenylpropanoyl)glycyl)-5-(*p*-tolyl)pyrrolidine-2-carboxylic Acid (AD015)

##### Step 1: *tert*-Butyl (*S*)-(2-(Acetylthio)-3-phenylpropanoyl)glycinate

TEA (2.7 mL, 0.026 mol) and 50% T_3_P in EtOAc (6 mL, 0.018 mol) were added sequentially to a solution of (*S*)-2-(acetylthio)-3-phenylpropanoic acid (1.4 g, 0.063 mol) in THF (25 mL) at 0 °C. After stirring for 30 min, *tert*-butyl glycinate (1.37g,0.008 mol) was added, and the mixture warmed to rt over 30 min before being quenched with water. Solids that precipitated were filtered, washed with hexanes, and dried under vacuum to give the title compound as a colorless solid. The solid was used in the next step without additional purification. Yield: 1.2 g; 57%.

##### Step 2: (*S*)-(2-(Acetylthio)-3-phenylpropanoyl)glycine

The preceding compound (1.2 g, 3.0 mmol) in ice-cooled trifluoracetic acid (5 mL) was stirred at rt for 15 min. The solvent was removed under vacuum to give a light brown solid that was used in Step 3 without purifying further. Yield: 1 g; 95%.

##### Step 3: Methyl (*S*)-2-((*tert*-Butoxycarbonyl)amino)-5-oxo-5-(*p*-tolyl)pentanoate (**5a**)

A THF (40 mL, 40 mmol) solution of 1 M *p*-tolylmagnesium bromide was added dropwise to a THF (250 mL) solution of 1-(*tert*-butyl) 2-methyl (*S*)-5-oxopyrrolidine-1,2-dicarboxylate (8.0 g, 32.09 mmol) at −40 °C under N_2_. After the mixture was warmed to 0 °C and stirred for 2 h, aq. NH_4_Cl (200 mL) was added. Extraction two times with EtOAc was followed by drying (Na_2_SO_4_) and removal of the solvent under 40 °C to afford **5a** as a white solid. The solid was used as such in the next step. Yield: 10.2 g; 92%. LCMS (M+H)^+^: 236.1 (fragment due to loss of *t*-butoxycarbonyl); ^1^H NMR (*d*_6_-DMSO); δ 7.8 (d, *J* = 8 Hz, 2H), 7.3 (d, *J* = 8 Hz, 2H), 4.05 (m, 1H), 3.6 (s, 3H), 1.9–3.1 (m, 2H), 2.37 (s, 3H), 2.0–2.1 (m, 1H), 1.8–1.9 (m, 2H), 1.3 (s, 9H).

##### Step 4: Methyl (*S*)-5-(*p*-Tolyl)-3,4-dihydro-2*H*-pyrrole-2-carboxylate

TFA (34.7 g, 0.304 mol) was added to the preceding compound (10.2 g, 30.4 mmol) dissolved in DCM at 0 °C, and the solution was warmed to rt for 16h. The solvent was removed by rotary evaporation, and the residue was dissolved in DCM, with the solvent again being removed. Purification by flash chromatography using EtOAc and pet ether as eluents gave the title compound as a yellow liquid. Yield: 6.5 g; 63%. LCMS, (M+H)^+^: 218.1; ^1^H NMR (*d*_6_-DMSO): δ 7.75 (d, *J* = 8 Hz, 2H), 7.25 (d, *J* = 8 Hz, 2H), 4.84 (t, *J* = 8 Hz, 1H), 3.0–3.2 (m, 1H), 1.9–3.0 (m, 1H), 2.36 (s, 3H), 2.2–2.3(m, 1H), 2.0–2.1 (m, 1H).

##### Step 5: Methyl (2*S*,5*R*)-5-(*p*-Tolyl)pyrrolidine-2-carboxylate (**6a**)

A mixture of the preceding compound (5g, 0.023 mol) and PtO_2_ (0.5 g, 10% w/w) in 50 mL MeOH was placed under a H_2_ atmosphere with stirring over 24 h. The mixture was rinsed through Celite with EtOAc. The filtrate was concentrated, and the residue was purified by flash chromatography using EtOAc and pet ether as eluents to afford **6a** as a yellowish gummy solid. Yield: 3.4 g; 70%. LCMS (M+H)^+^: 220.1; ^1^H NMR (*d*_6_-DMSO): δ 7.3 (d, *J* = 8 Hz, 2H), 7.1 (d, *J* = 8 Hz, 2H), 4.1 (m, 1H), 3.8 (m, 1H), 3.66 (s, 3H), 3.0 (br s, 1H), 2.27 (s, 3H), 2.0–2.1 (m, 2H), 1.9–2.0 (m, 1H), 1.5 (m, 1H).

##### Step 6: Methyl (2*S*,5*R*)-1-(((*S*)-2-(Acetylthio)-3-phenylpropanoyl)glycyl)-5-(*p*-tolyl)pyrrolidine-2-carboxylate (**7b**)

TEA (3.5 mL, 0.034 mol) and a 50% solution of T_3_P in EtOAc (6 mL, 0.018 mol) were added sequentially to an ice-cooled THF (10 mL) solution of (*S*)-(2-(acetylthio)-3-phenylpropanoyl)glycine (1 g, 3.0 mmol, from Step 2), and the mixture was stirred for 30 min. **6a** (1 g, 4.0 mol) was added, and the reaction mixture was warmed to rt over 30 min. The reaction mixture was quenched with water, and the precipitated solid was filtered and washed with hexane before being dried under a vacuum to afford **7b** as a light brown solid. The solid was used in the subsequent step as such. Yield: 1 g; 58%.

##### Step 7: (2*S*,5*R*)-1-(((*S*)-2-Mercapto-3-phenylpropanoyl)glycyl)-5-(*p*-tolyl)pyrrolidine-2-carboxylic Acid (AD015)

LiOH (0.47g, 0.011 mol) was added to a solution of **7b** (1 g, 2.0 mmol) in 10 mL of 1:1 THF/water, and the mixture was stirred at rt for 2 h. The reaction mixture was diluted with water and extracted several times with EtOAc. The EtOAc extracts were dried (Na_2_SO_4_) and concentrated. The residue was purified by flash chromatography, followed by preparative HPLC to afford AD015 as an off-white solid. Yield: 130 mg; 15%. LCMS, (M+H)^+^: 427.1; ^1^H NMR (*d*_6_-DMSO): δ 12.3, (br s., 1H), 7.8 (m, 1H), 8.0 (t, *J* = 5 Hz, 1H), 7.5(m, 1H), 7.0–7.4 (m, 7H), 5.1 (m, 1H), 4.3–4.4 (m, 1H), 3.85–3.95 (m, 1H), 3.8–3.9 (m, 1H), 3.65–3.8 (m, 1H), 3.0–3.4 (m, 2H), 2.7–2.8 (m, 1H), 2.0–2.4 (m and s, total of 6H), 1.75–1.9 (m, 2H). ^13^C NMR (*d*_6_-DMSO): δ 173.7, 172.1, 168.3, 140.3, 139.1, 136.8, 129.7, 129.5. 128.6, 126.8, 126.6, 61.4, 60.9, 42.7, 42.5, 41.9, 41.6, 36.3, 27.4, 21.1.

#### Synthesis of ((*S*)-2-Mercapto-3-phenylpropanoyl)-l-lysyl-l-tryptophan (AD016)

##### Step 1: *tert*-Butyl*N*^2^-((*S*)-2-(Acetylthio)-3-phenylpropanoyl)-*N*^6^-((benzyloxy)carbonyl)-l-lysinate

TEA (1.86 mL, 13.4 mol) and a solution of 50% T_3_P in EtOAc (5.7 mL, 8.9 mmol) were added in sequence to an ice-cooled THF (10 mL) solution of (*S*)-2-acetylthio-3-phenylpropionic acid (1 g, 0.004.5 mmol). After the mixture was stirred at 0 °C for 30 min, *tert*-butyl (2*S*)-2-amino-6-{[(benzyloxy)carbonyl]amino}hexanoate (1.95 g, 5.8 mmol) was added, with the reaction mixture being warmed to rt over 30 min. After quenching with water, and solids that precipitated were filtered, washed with hexane, and dried to afford the title compound as a solid. The solid was propagated to the next step without additional purification. Yield: 1.1 g; 45%.

##### Step 2: *N*^2^-((*S*)-2-(Acetylthio)-3-phenylpropanoyl)-*N*^6^-((benzyloxy)carbonyl)-l-lysine

A TFA (10 mL) solution of the preceding compound (1.1 g, 2.0 mmol) was stirred at rt for 15 min. The solvent was removed via rotary evaporation, and the residue was dried under high vacuum, giving the title compound as a colorless solid. No further purification was carried out before progressing to the next step. Yield: 0.6 g; 61%.

##### Step 3: Methyl*N*^2^-((*S*)-2-(Acetylthio)-3-phenylpropanoyl)-*N*^6^-((benzyloxy)carbonyl)-l-lysyl-l-tryptophanate (**9**)

TEA (0.51 mL, 0.0037 mol, 3 equiv) and a solution of 50% T_3_P in EtOAc (1.6 mL, 2.47 mmol) were added sequentially to an ice-cooled solution of the prior compound (0.6 g, 1.23 mol) in THF (10 mL) at 0 °C. After stirring 30 min, methyl-l-tryptophanate hydrochloride (0.41 g, 1.6 mmol) was added, and the mixture was warmed to ambient temperature. Quenching water led to solids precipitating. The solids were filtered, washed with hexanes, and dried in vacuo to give **9** as a light brown solid. The solid was used in the next step without any further treatment. Yield: 0.6 g; 70%.

##### Step 4: *N*^6^-((Benzyloxy)carbonyl)-*N*^2^-((*S*)-2-mercapto-3-phenylpropanoyl)-l-lysyl-l-tryptophan

LiOH (0.183 g, 4.4 mmol) was added to a stirred solution of **9** (0.6 g, 0.87 mmol) in a mixture of THF (5 mL) and water (5 mL). After 2 h stirring at ambient temperature, the mixture was diluted with water and extracted with several times with EtOAc. The EtOAc layers were dried (Na_2_SO_4_) and concentrated to give the title compound as a brownish solid. Yield: 0.4 g; 72%.

##### Step 5: ((*S*)-2-Mercapto-3-phenylpropanoyl)-l-lysyl-l-tryptophan (AD016)

A solution of the preceding compound (0.4 g, 6.3 mmol) in TFA (2 mL) was heated at 70 °C for 20 min. TFA was removed by rotary evaporation. The residue was purified by preparative HPLC followed by chiral purification to afford AD016 (TFA salt) as a white solid. Yield: 0.06 g, 15%. LCMS (M+H)^+^: 497.2; ^1^H NMR (*d*_6_-DMSO): −12.7, (br s., 1H), 10.9 (s, 1H), 8.2 (d, *J* = 7 Hz, 1H), 8.1 (d, *J* = 8 Hz, 1H), 7.7 (m, 2H), 7.5 (d, *J* = 8 Hz, 1H), 7.3 (d, *J* = 8 Hz, 1H), 7.0–7.2 (m, 6H), 6.9–7.0 (m, 1H), 4.4–4.5 (m, 1H), 4.3–4.4 (m, 1H), 3.7 (q, *J* = 6 Hz, 1H), 3.1–3.2 (m, 2H), 3.0–3.1 (m, 1H), 2.6–2.8 (m, 3H), 1.65 (m, 1H), 1.5 (m, 3H), 1.3 (m, 2H). ^13^C NMR (*d*_6_-DMSO): δ 173.6, 172.1, 171.7, 139.1, 136.5, 129.5, 129.2, 128.6, 127.6, 126.8, 124.1, 121.4, 118.8, 118.6, 111.8, 110.1, 53.4, 52.4, 42.8, 41.4, 27.5, 27.2, 22.4.

### Compound Dissolution and Stability

LisW and compounds AD014–AD016 were synthesized by CRO, Syngene. Omapatrilat was purchased from Sigma-Aldrich. Compound stock solutions at a concentration of 5 or 10 mM were prepared in DMSO for all compounds except LisW which was dissolved in dH_2_O. For thiol containing compounds, fresh compound stocks were prepared in DMSO on the day of the experiment and immediately diluted in assay buffer to working stock concentrations to limit disulfide bond formation. Compound integrity was monitored at a 50 μM concentration by HPLC to ensure that disulfide bond formation was <5% during the course of the experiments.

### ACE Inhibition Assays

Purified fully glycosylated human cACE and nACE proteins^36,37^ were used in fluorogenic end point activity assays utilizing Cbz-Phe-His-Leu (Z-FHL, Bachem Ltd., nACE *K*_m_ = 600 μM; cACE *K*_m_ = 60 μM) as the substrate as previously described.^32^ Final reactions contained ∼1 nM nACE/cACE and 0.5 mM Z-FHL. IC_50_ values were calculated from *n* ≥ 2 independent experiments.

### NEP Inhibition Assays

A purified human NEP ectodomain was used in continuous activity assays utilizing MCA-RPPGFSAFK(Dnp)–OH peptide (R&D Systems; NEP *K*_m_ = 7 μM) as the substrate as previously described.^32^ Final reactions contained 0.4 nM NEP and a 5 μM substrate. IC_50_ values were calculated from *n* ≥ 2 independent experiments.

### X-ray Protein Crystallography

As reported previously^36,38^ minimally glycosylated nACE and cACE (N389 and G13, respectively) were generated by expression in cultured mammalian CHO cells and purified to homogeneity (assessed by SDS-PAGE and were shown to be >95% pure). ACE was preincubated with the ligands for 1 h (at room temperature for nACE and on ice for cACE) using a 4:1 v/v ratio of protein (5 and 8 mg mL^–1^ nACE and cACE, respectively, in 50 mM HEPES, pH 7.5, 0.1 mM PMSF) and 1 mM AD014, AD015, or AD016 (10 mM stocks in DMSO, diluted to 1 mM with water). Crystals were grown using hanging drops of 1 μL of the protein-inhibitor complex mixed with 1 μL of reservoir solution. Standard Molecular Dimensions Morpheus A9 condition (30% PEG 550 MME/PEG 20000, 0.1 M Tris/Bicine pH 8.5 and 60 mM divalent cations) was used for nACE. The cACE complexes crystallized in 0.1 M MIB buffer pH 4.0 and 5% v/v glycerol, with varying amounts of PEG 3350 (16% v/v for AD014 and AD015, and 15% v/v for AD016).

X-ray diffraction data were collected on stations i04 (nACE in complex with AD014 and AD015, and cACE-AD016) and i24 (cACE in complex with AD014 and AD015) at the Diamond Light Source (Didcot, UK). During data collection, the crystals were kept at 100 K using a nitrogen stream. Images were collected using detectors PILATUS-6M-F and Eiger2 XE 16 M (i04) and PILATUS3 6 M (i24) (Dectris, Switzerland). Raw data images were indexed and integrated with DIALS,^39^ followed by scaling using AIMLESS^40^ from the CCP4 suite.^41^ Initial phases were obtained by molecular replacement method with PHASER^42^ using N389-nACE PDB code 6F9 V^43^ and G13-cACE PDB code 6F9T(43) as search models for nACE and cACE, respectively. Further refinement was carried out initially using REFMAC5^44^ and then Phenix,^45^ with COOT^46^ used for rounds of manual model building. Ligand and water molecules were added based on electron density in the mFo-DFc Fourier difference map. MolProbity^47^ was used to validate the structures. All crystallographic data statistics are summarized in Table 2. All figures showing the crystal structures were generated using CCP4 mg,^48^ and schematic binding interactions are displayed using LigPlot+.^49^

### In Silico Docking

The crystal structures of human nACE (PDB ID 6H5X) and NEP (PDB ID 6SUK) in complex with omapatrilat were used as initial model structures for docking of AD016 into nACE and AD014, AD015, and AD016 into NEP. The model structures were prepared for docking studies using the Protein Preparation Wizard tool in Maestro (Schrodinger, LLC, New York, USA). 3D models of inhibitors were prepared for modeling using Maestro's LigPrep module. Initial binding models of AD016 in nACE and AD014, AD015, and AD016 in NEP were generated by prime minimization of the ligand and the protein within 6 Å of the ligand, using the OPLS4 force field, the variable-dielectric generalized Born (VSGB) solvation model for water. Final binding models were generated using MM-GBSA minimization of the ligand and protein binding site within 6 Å of the ligand. Modeling figures were produced using PyMOL, Schrodinger, LLC.

## Supporting Information Available

Supplementary enzyme kinetic methods and results (PDF)
In silico docking files of nACE-AD016, NEP-AD014, NEP-AD015, and NEP-AD016 (ZIP)
Molecular formula strings (CSV)

## Accession Codes

The atomic coordinates and structure factors have been deposited in the RCSB Protein Data Bank (www.rcsb.org/pdb) with accession numbers 9H1A (nACE-AD014), 9H1B (nACE-AD015), 9H1C (cACE-AD014), 9H1D (cACE-AD015), and 9H1E (cACE-AD016). The atomic coordinates and experimental data will be released upon article publication.

## Abbreviations Used

ACE: angiotensin-1 converting enzyme
ARBs: angiotensin receptor blockers
Ac-SDKP: *N*-acetyl-Ser-Asp-Lys-Pro
CHO: Chinese hamster ovary cells
DMSO: dimethyl sulfoxide
Dnp: dinitrophenyl
NEP: neprilysin
PDB: Protein Data Bank
RAS: renin-angiotensin system
ZBG: zinc binding group
