# Supplementary material for: Design of Novel Mercapto-3-phenylpropanoyl Dipeptides as Dual Angiotensin-Converting Enzyme C–Domain-Selective/Neprilysin Inhibitors
Source: J Med Chem. 2025 Apr 1;68(7):7720–36. doi: 10.1021/acs.jmedchem.5c00329 (PMC11997989; doi:10.1021/acs.jmedchem.5c00329)
Supplement: Supplementary file 1 — jm5c00329_si_001.pdf [file jm5c00329_si_001.pdf]

Supplementary Information

The Design of Novel Mercapto-3-Phenylpropanoyl Dipeptides as Dual Angiotensin-Converting Enzyme C-Domain–Selective/Neprilysin Inhibitors

Gyles E. Cozier[±], Lauren B. Coulson[†,‡], Charles J. Eyermann[⊥], Gregory S. Basarab[⊥], Sylva L. Schwager[†,‡], Kelly Chibale[†,⊥,§,‖], Edward D. Sturrock[†,‡,*], K. Ravi Acharya[±,*]

[±]Department of Life Sciences, University of Bath, Claverton Down, Bath BA2 7AY, United Kingdom

[†]Institute of Infectious Disease and Molecular Medicine, University of Cape Town, Observatory 7925, South Africa

[‡]Department of Integrative Biomedical Sciences, University of Cape Town, Observatory 7925, South Africa

[⊥]Drug Discovery and Development Centre (H3D), University of Cape Town, Rondebosch 7701, South Africa

[§]Department of Chemistry, University of Cape Town, Rondebosch 7701, South Africa

[‖]South African Medical Research Council Drug Discovery and Development Research Unit, University of Cape Town, Rondebosch 7701, South Africa

*Joint corresponding authors. E-mail: Edward D. Sturrock edward.sturrock@uct.ac.za ; K. Ravi Acharya bsskra@bath.ac.uk

Table of Contents

Supplementary data

^{1}H, ^{13}C NMR and HPLC-MS for AD014, AD015 and AD016

^{1}H NMR (295.9 °K)– AD014……………………………………………S3

^{1}H NMR (356.2 °K)– AD014……………………………………………S3

^{13}C NMR – AD014…………………………………………………S4

HPLC-MS – AD-014………………………………………………….S4

^{1}H NMR – AD015…………………………………………………..S5

^{13}C NMR – AD015…………………………………………………S5

HPLC-MS – AD-015………………………………………………….S6

^{1}H NMR – AD016…………………………………………………..S6

^{13}C NMR – AD016……………………………………...……..……S7

HPLC-MS – AD-016………………………………………………….S7

Supplementary Results

Mode of inhibition studies and challenges determining inhibition constants for mercaptoacyl dipeptides

Table S1: Substrates used for inhibition assays………………………S8

Figure S1……………………….…………………………………S10

Figure S2……………………….…………………………………S11

Figure S3……………………….…………………………………S12

Figure S4……………………….…………………………………S13

Supplementary Methods

Jump dilution nACE and cACE assays ………………………………..…S13

Enzyme Data Analysis……………………………….…………………S13

Supplementary data

^{1}H,^{13}C NMR and HPLC-MS for AD014, AD015 and AD016

^{1}H NMR (295.9 °K)– AD014

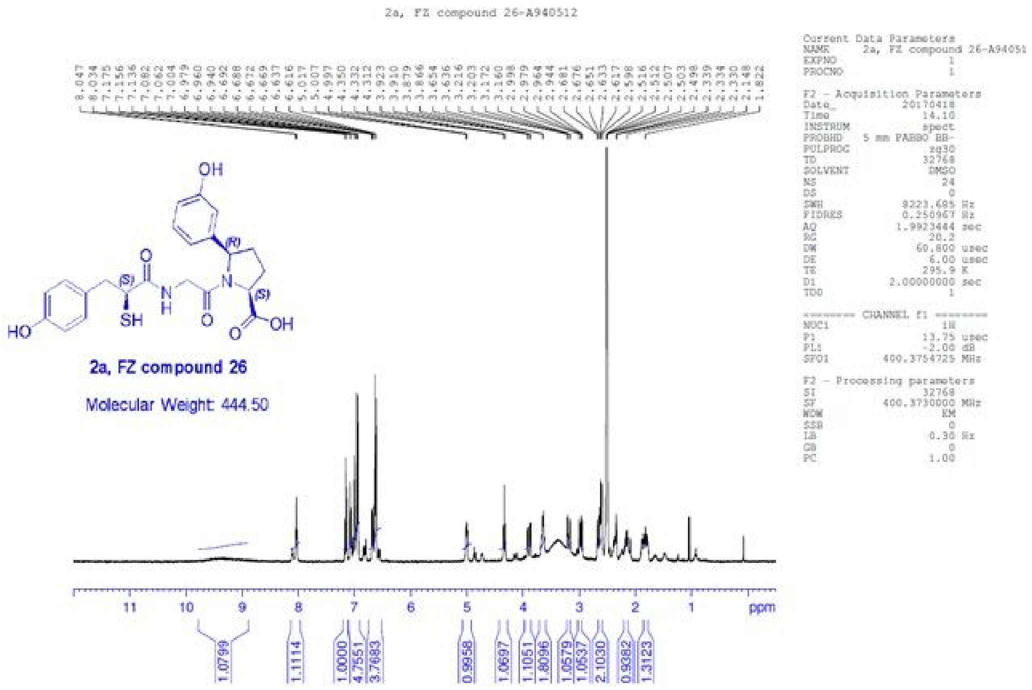

^{1}H NMR (356.2 °K)– AD014

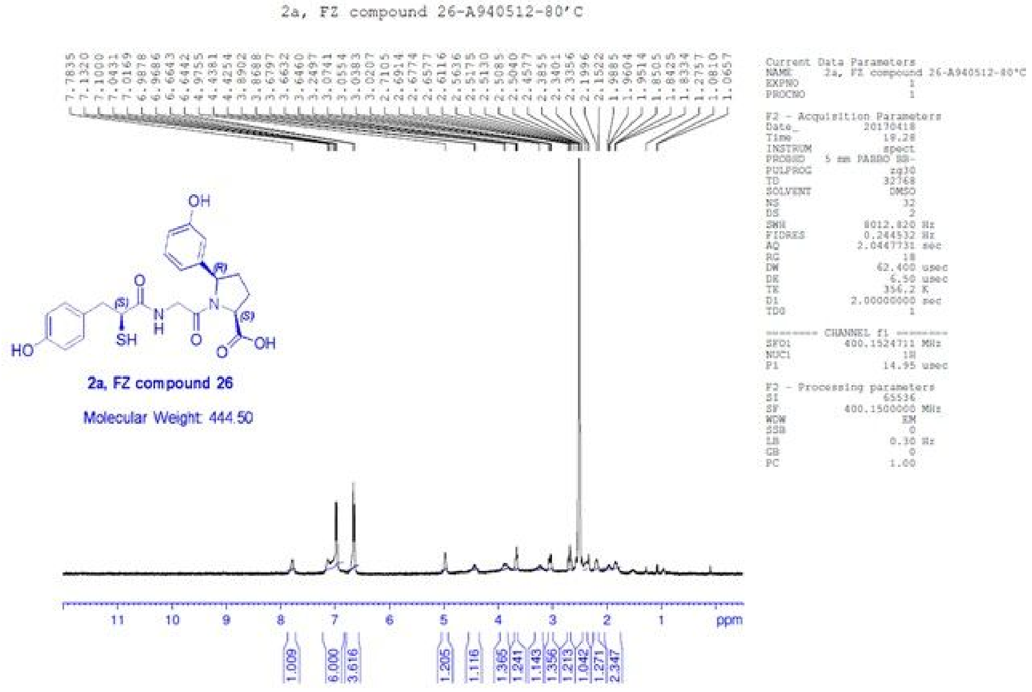

^{13}C NMR – AD014

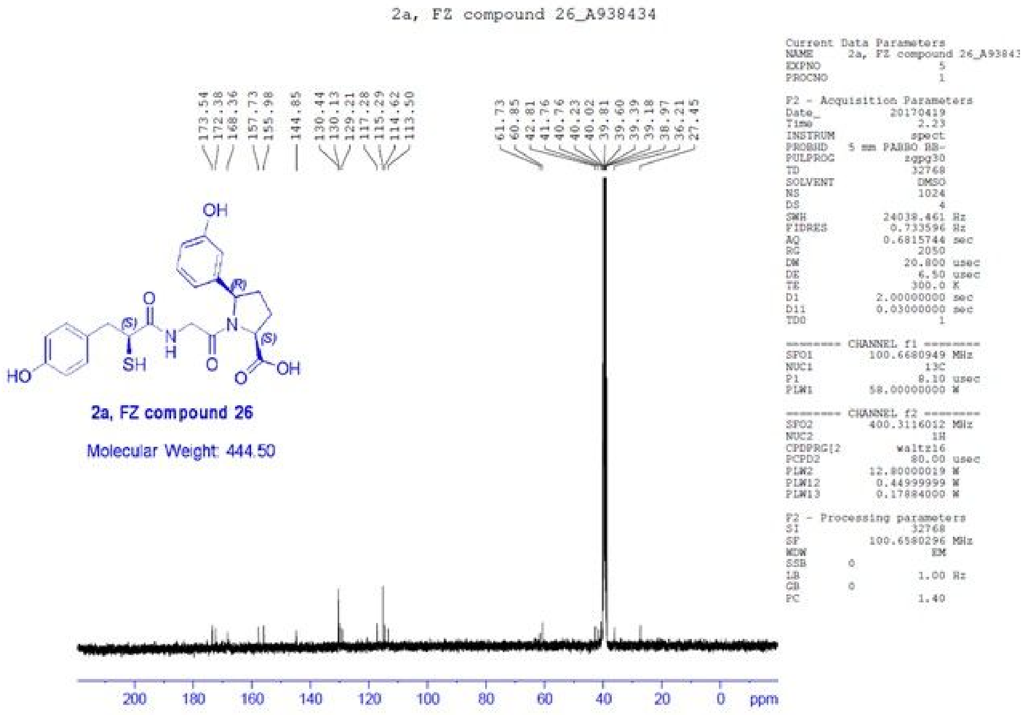

HPLC-MS – AD014

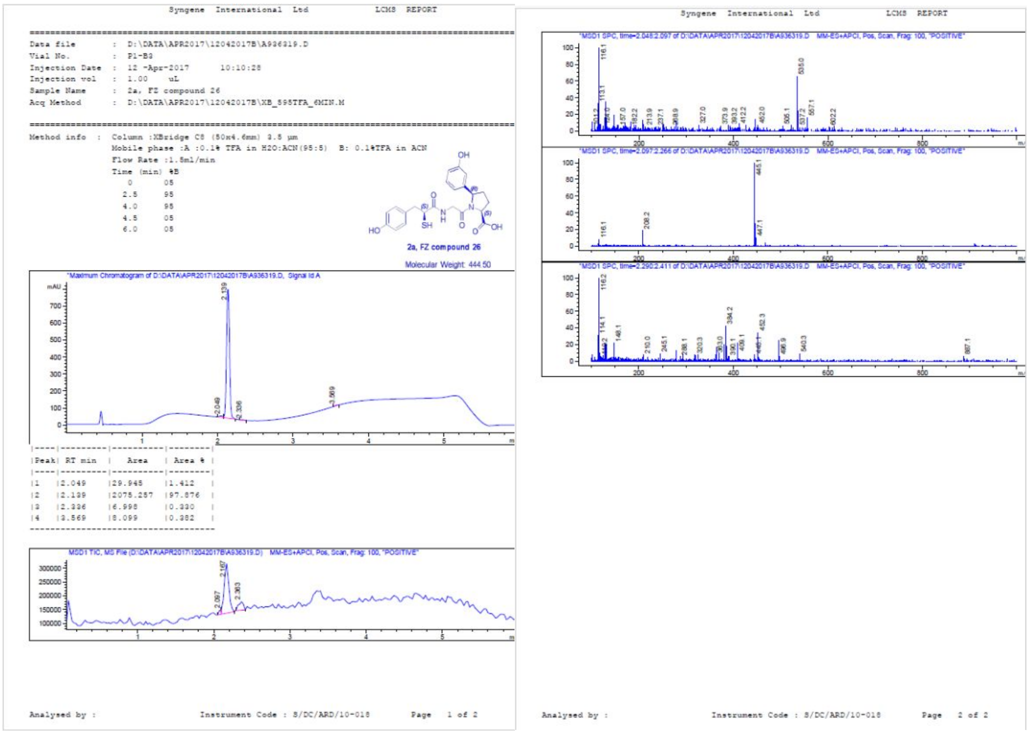

Peak	RT min	Area	Area %
1	2.049	29.945	1.412
2	2.139	2075.257	97.876
3	2.336	6.998	0.330
4	3.569	8.099	0.382

^{1}H NMR – AD015

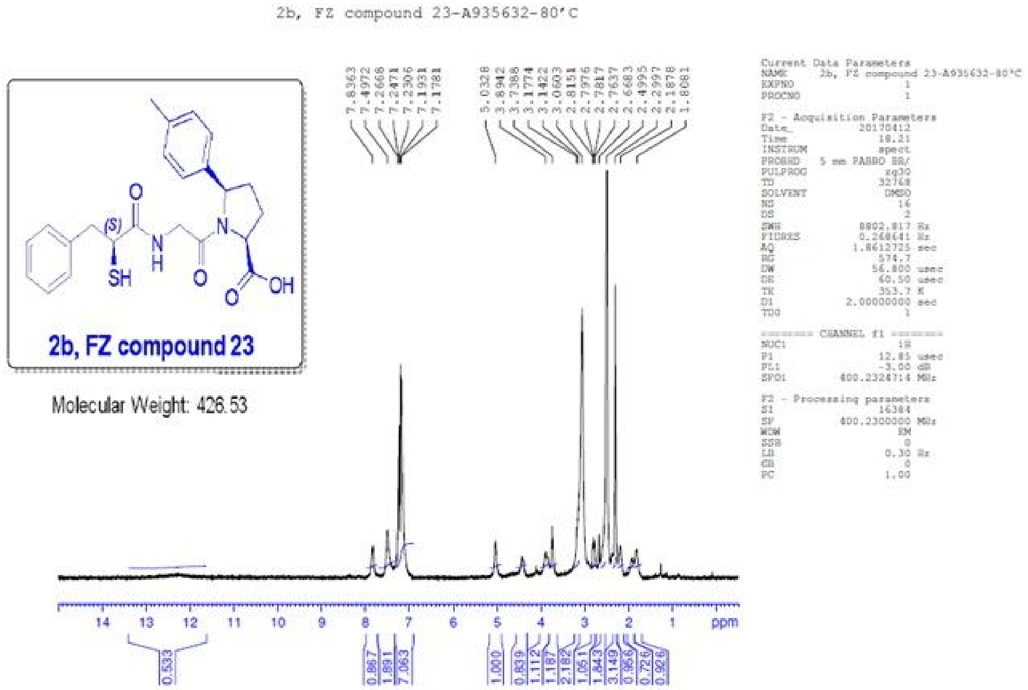

^{13}C NMR – AD015

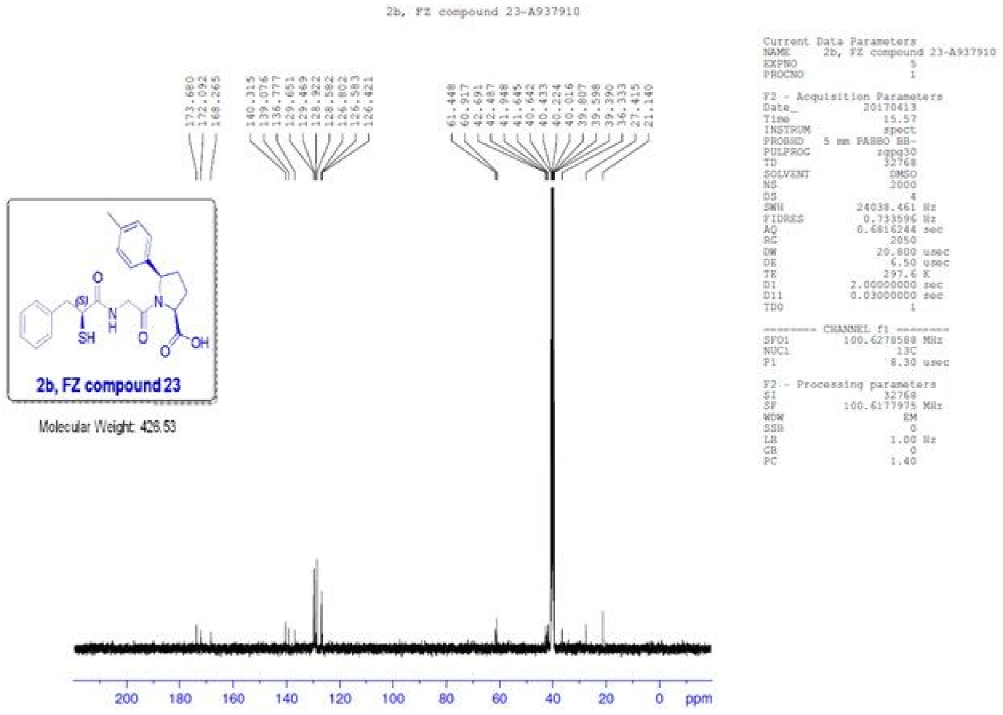

HPLC-MS – AD015

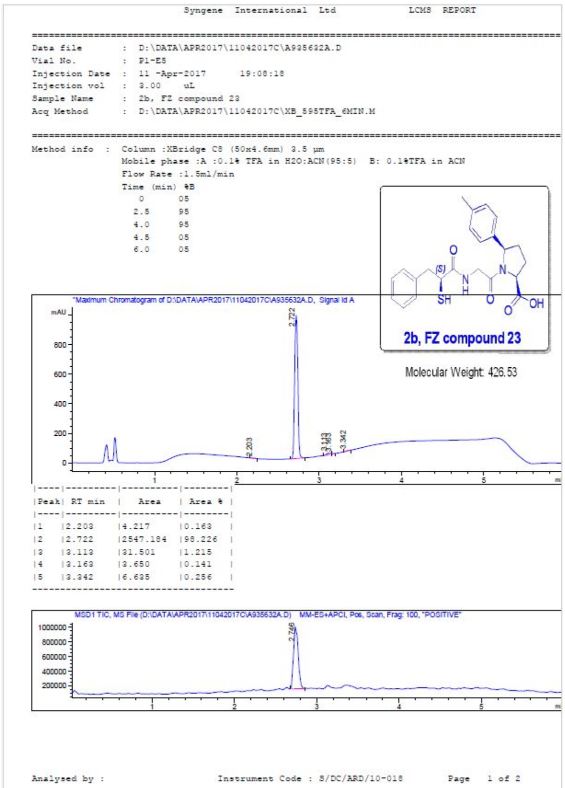

Syngene International Ltd LCMS REPORT

Data file : D:\DATA\APR2017\11042017C\A935632A.D
Vial No. : P1-E5
Injection Date : 11 -Apr-2017 19:08:18
Injection vol : 3.00 uL
Sample Name : 2b, FZ compound 23
Acq Method : D:\DATA\APR2017\11042017C\XB_595TFA_6MIN.M

Method info : Column :XBridge C8 (50x4.6mm) 3.5 µm
Mobile phase :A :0.1% TFA in H2O:ACN(95:5) B: 0.1%TFA in ACN
Flow Rate :1.5ml/min

Time (min)	%B
0	05
2.5	95
4.0	95
4.5	05
6.0	05

Peak	RT min	Area	Area %
1	2.203	4.217	0.163
2	2.722	2547.184	98.226
3	3.113	31.501	1.215
4	3.163	3.650	0.141
5	3.342	6.635	0.256

Analysed by : Instrument Code : S/DC/ARD/10-018 Page 1 of 2

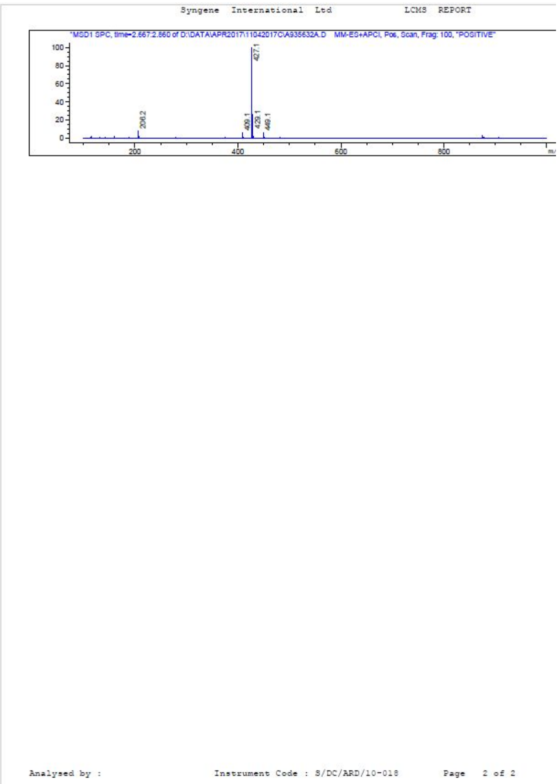

Analysed by : Instrument Code : S/DC/ARD/10-018 Page 2 of 2

^{1}H NMR – AD016

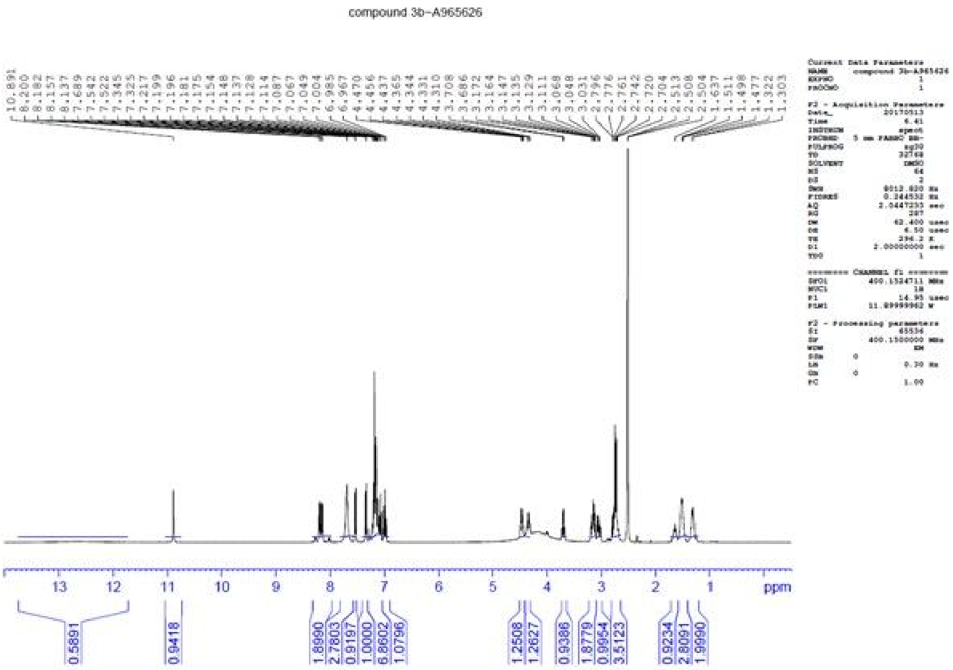

^{13}C NMR – AD016

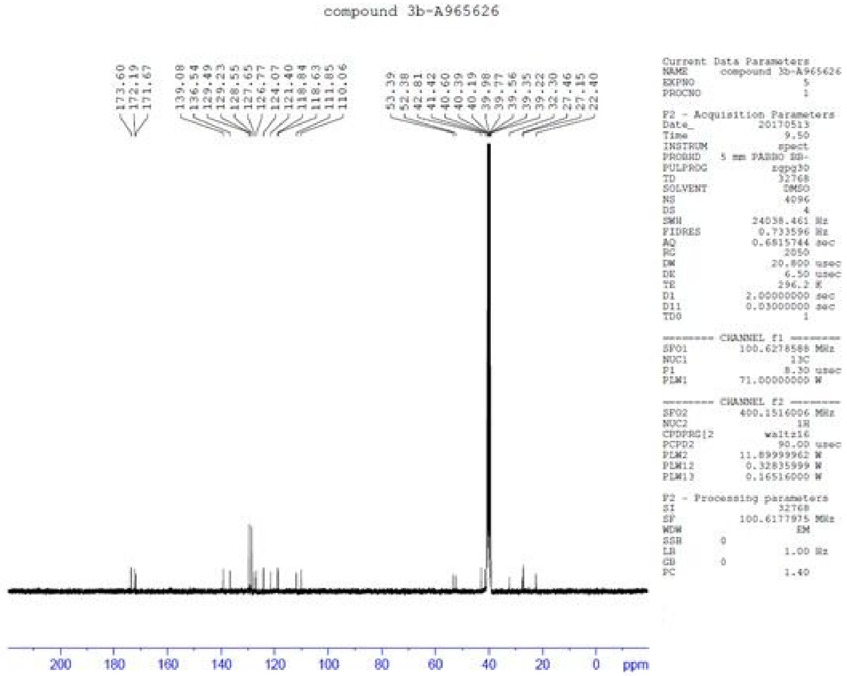

HPLC-MS – AD016

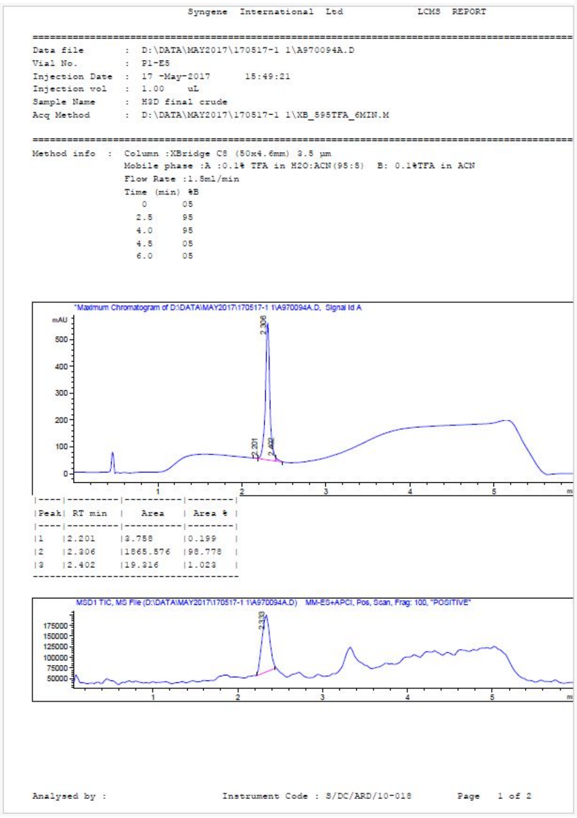

Syngene International Ltd LCMS REPORT

Data file : D:\DATA\MAY2017\170517-1 1\A970094A.D
Vial No. : P1-E5
Injection Date : 17 -May-2017 15:49:21
Injection vol : 1.00 uL
Sample Name : H3D final crude
Acq Method : D:\DATA\MAY2017\170517-1 1\XB_595TFA_6MIN.M

Method info : Column :XBridge C8 (50x4.6mm) 3.5 µm
Mobile phase :A :0.1% TFA in H2O:ACN(95:5) B: 0.1%TFA in ACN
Flow Rate :1.5ml/min

Time (min)	%B
0	05
2.5	95
4.0	95
4.5	05
6.0	05

Peak	RT min	Area	Area %
1	2.201	3.758	0.199
2	2.306	1865.576	98.778
3	2.402	19.316	1.023

Analysed by : Instrument Code : S/DC/ARD/10-018 Page 1 of 2

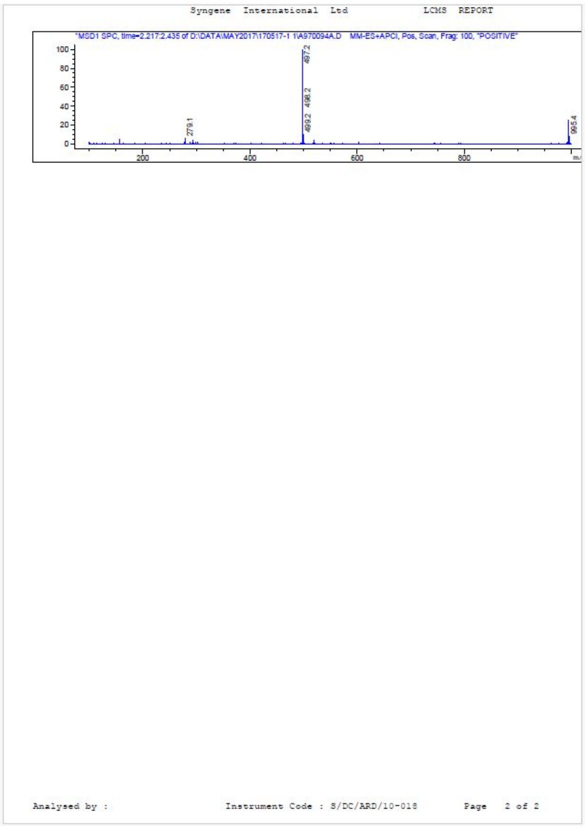

Syngene International Ltd LCMS REPORT

Analysed by : Instrument Code : S/DC/ARD/10-018 Page 2 of 2

Supplementary Results

Mode of inhibition studies and challenges determining inhibition constants for mercaptoacyl dipeptides

For an accurate comparison of the potency of the inhibitors between enzymes it is preferable to determine inhibition constants (K_i values) rather than IC_{50} values as true K_i values are independent of the enzyme concentration, the substrate concentration and the affinity of the substrate for the enzyme (K_m). Typically, for competitive inhibitors, inhibition constants can be calculated from IC_{50} values determined from IC_{50} log plots using Equation 1 which factors in the K_m of the substrate and the substrate concentration used in the assay, provided the assay conditions conform to the classical inhibition model for competitive inhibition. The K_m values for the substrates used in this study are shown in Table S1.

$$K_i = \frac{IC_{50}}{\left(1 + \frac{[S]}{K_m}\right)} \qquad \textbf{Eq 1}$$

Where K_i in the inhibition constant, IC_{50} is the inhibitor concentration giving 50% decrease in enzyme activity, [S] is the substrate concentration and K_m is the Michaelis-Menten constant of the substrate for the enzyme.

Table S1: Substrates used for inhibition assays

Enzyme	Substrate	K_m (μM)
ACE N-domain	Z-FHL	600
ACE C-domain	Z-FHL	60
NEP	MCA-RPPGFSAFK(Dnp)-OH	7

Under classical inhibition conditions E_t (total enzyme concentration) << K_i (inhibition constant of the compound tested) and consequently the IC_{50} will be independent of the enzyme concentration. For potent inhibitors, like the omapatrilat and the mercaptoacyl dipeptides in this study, it is not possible to carry out the reactions at sufficiently low enzyme concentrations to conform to the conditions required for Michaelis-Menten kinetics ($E_t << K_i$). In this case, the lowest theoretical IC_{50} that can be obtained from an IC_{50} log plot is $E_t/2$. Under tight-binding conditions, where $E_t \approx K_i$, the data should be fitted to the Morrison equation (Equation 2) which corrects for the effect of enzyme concentration. The total enzyme concentration can be constrained to a constant or it can be determined based on the fit of curve to the data – as for an active-site titration - provided there are sufficient data points in the titration portion of the

curve. If the E_t determined by the equation is very different from the E_t used in the experiment it may indicate that the either the concentration of the inhibitor or the concentration of the enzyme is incorrect, or that the model does not accurately explain the data. The apparent K_i (K_i^{app}) obtained from this equation can then be converted to K_i as for an IC_{50} value using Equation 1 provided the substrate competes with the inhibitor for the active site.

$$V_i = V_0\left(1 - \frac{(E_t+[I]+K_i^{app}) - \sqrt{(E_t+[I]+K_i^{app})^2 - 4E_t[I]}}{2E_t}\right) \qquad \textbf{Eq 2}$$

Where V_o is activity in absence of inhibitor, V_i is activity in presence of inhibitor, [I] is inhibitor concentration and E_t is total enzyme concentration.

Due to the slow binding of many protease inhibitors, inhibitors are typically pre-incubated with the enzyme prior to the addition of substrate to ensure that inhibition constants are measured under steady-state conditions. Accordingly, if an inhibitor forms a very tight complex with the enzyme resulting in a very slow off-rate, the inhibitor may not dissociate in the time of the experiment.

Determining the *K*i values for the mercaptoacyl inhibitors was challenging due to the chemical instability of the thiol group (although this was controlled by making up fresh stock before each experiment and diluting them immediately), the high potency, and the slow off rates observed in some cases, particularly for cACE. In many cases, the data for these compounds did not fit well to any of the conventional inhibition models, making it difficult to interpret and compare data between enzymes and compounds systematically. This is exemplified by the data for **AD016**. Figure S1 shows Michaelis-Menten inhibition kinetics for inhibition of NEP, nACE and cACE. **AD016** showed competitive inhibition for NEP and the **nACE** but non-competitive inhibition for **cACE**, suggesting that the substrate was unable to compete with the inhibitor for the cACE active site and that the inhibition was essentially irreversible during the time frame of the experiment. The K_i values for the cACE determined in from this model were also inconsistent and were influence by the amount of enzyme used to the assay, again indicative of tight-binding conditions (Figure S1 C and D).

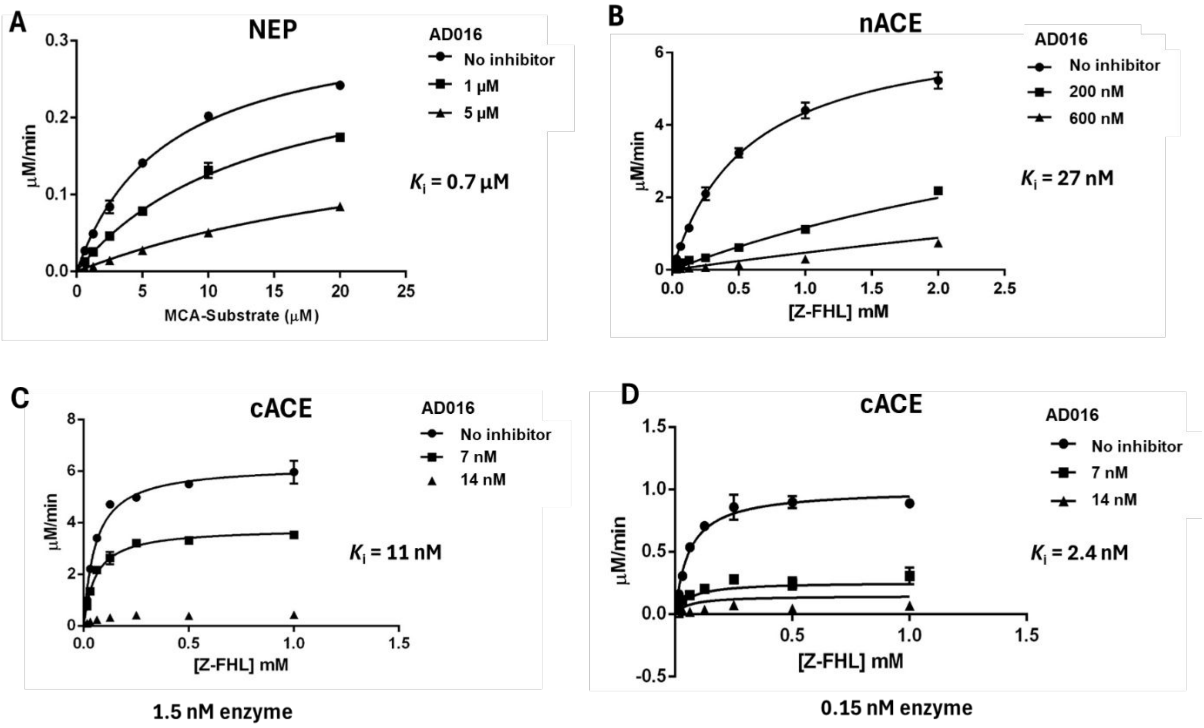

Figure S1: Inhibition of NEP (A), nACE (B) and cACE (C and D) by compound **AD016**. Enzyme and inhibitor were pre-incubated for 15 min prior to the addition of substrate. Final substrate, inhibitor and enzyme concentration are indicated on the plots. A competitive inhibition model fitted best to the NEP and nACE data (A and B); while a non-competitive inhibition model fitted best to the cACE data (C and D). The effect of different enzyme concentrations on the K_i values determined from this model for cACE inhibition is illustrated by graphs C and D.

The apparent non-competitive or "irreversible" binding of **AD016** to cACE was further confirmed by jump dilution inhibition assays which showed that inhibitor **AD016** readily dissociates from nACE when diluted but remains tightly bound to the cACE – even after 3 hours (Figure S2), further indicating tight binding conditions.

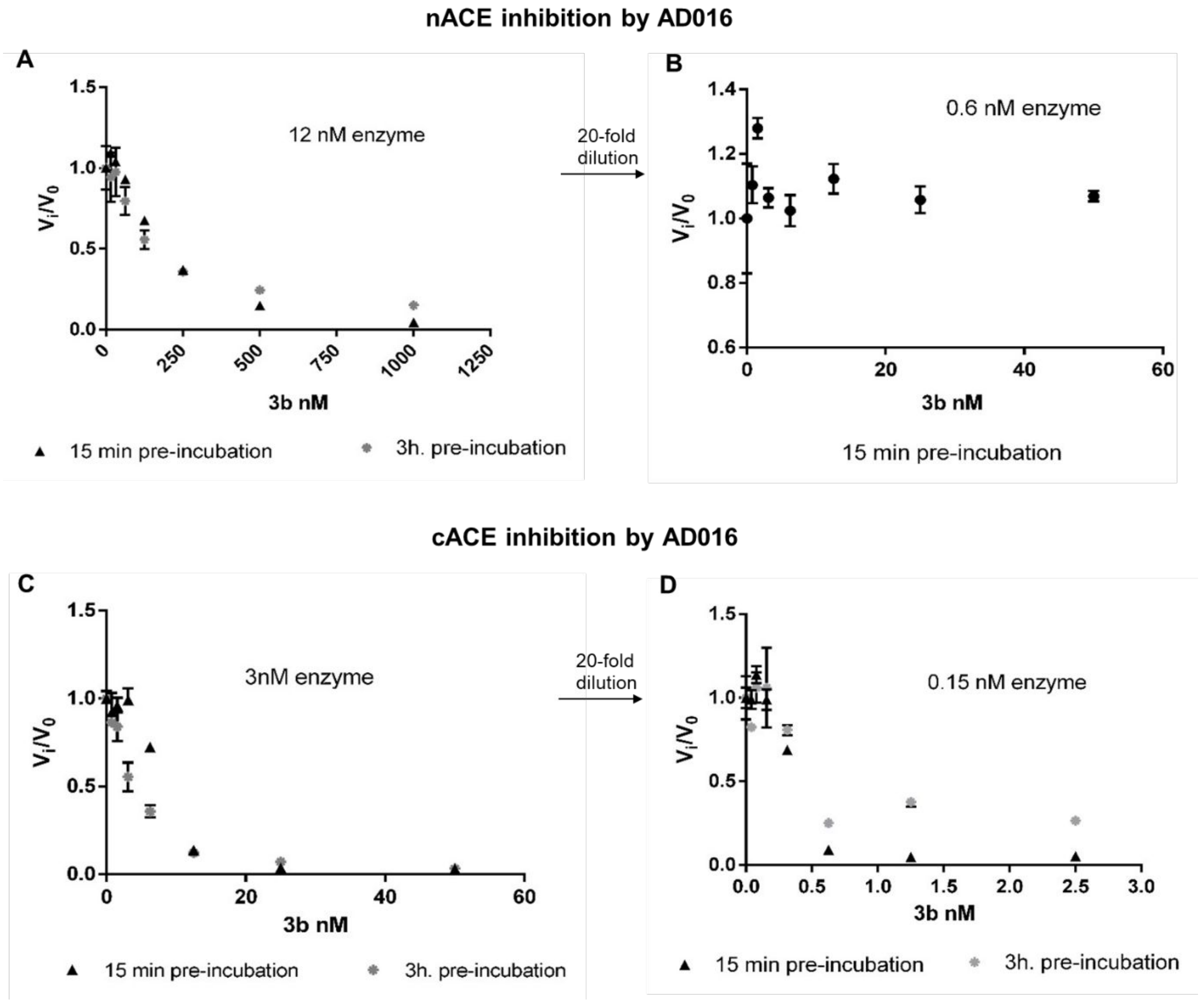

Figure S2: Jump dilution experiment forAD016. A) Enzyme and inhibitor were pre-incubated for 15 min and then an aliquot of each was removed and diluted 20-fold in assay buffer. Activity assays were performed on both the undiluted (A and C) and diluted (B and D) enzyme-inhibitor mixes immediately and ~3h. later. Reaction was initiated with Z-FHL substrate (1mM final concentration). Final enzyme and inhibitor concentrations are indicated on the graphs.

Figure S3 shows IC_{50} plots and Morrison plots for inhibition of the nACE and cACE by **AD016**. The IC_{50} curves show that activity drops off rapidly, as shown by the high negative Hill coefficients, particularly for cACE, with only a few points on the slope of the curve, as seen for omapatrilat (Figure S3 A and B). The Morrison equation was fitted to the same data but gave a poor fit when E_t was constrained to the enzyme concentration used in the assay and an unrealistic E_t if this value was left unconstrained (Figure S3 C & D). The obvious explanation for the high E_t predicted by the model is that the inhibitor concentration is much lower than expected. However, the data for the nACE and cACE does not support this explanation since the nACE data would imply the inhibitor concentration is ~350-fold lower than expected while the cACE data implies it is 40-fold lower than expected. The same inhibitor dilution series was used for both assays. In addition, a subsequent experiment showed that a 5-fold decrease in

enzyme concentration did not correspond to a 5-fold decrease (or any decrease) in the E_t determined by the Morrison plot, confirming that this model could not reliably explain the data Figure S4).

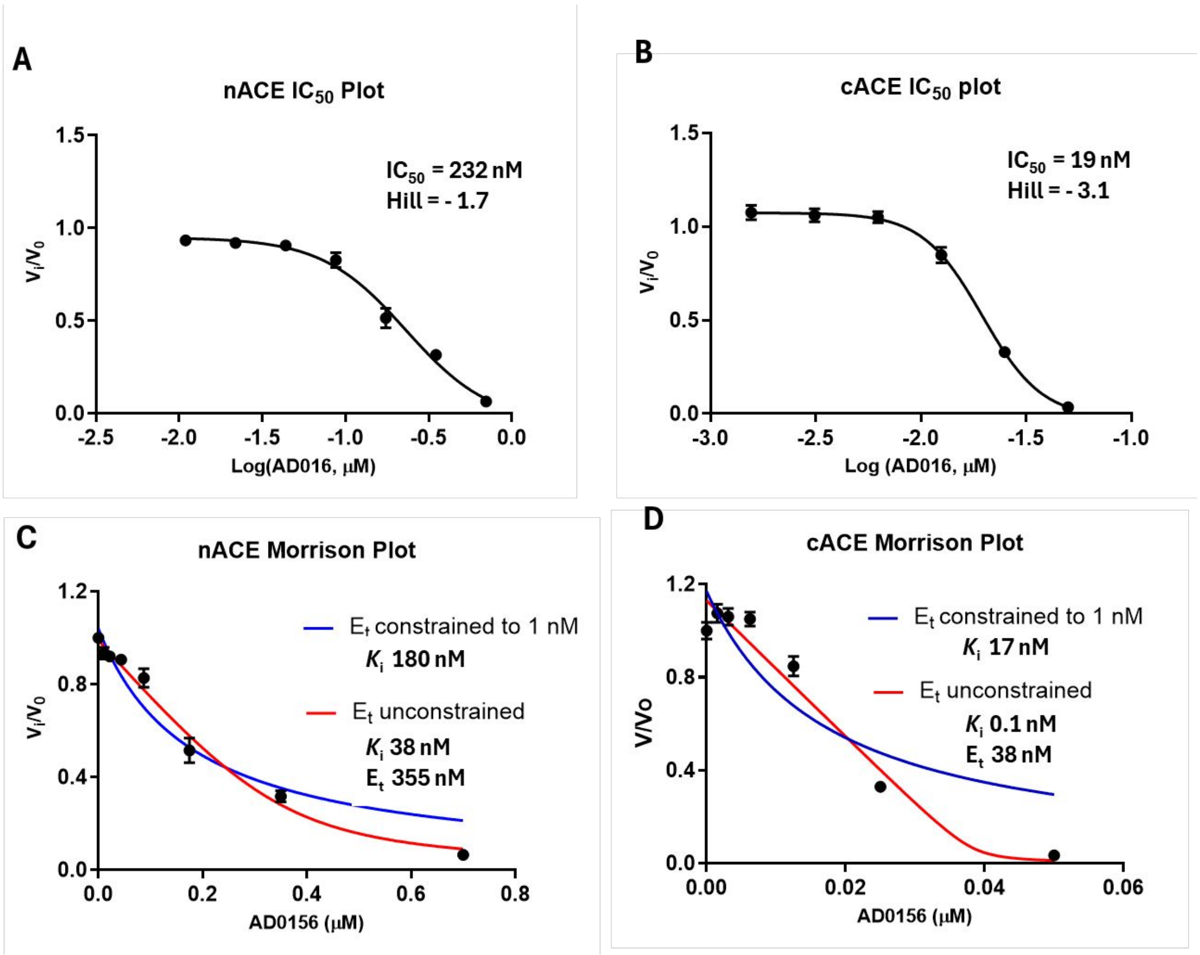

Figure S3: Inhibition of nACE and cACE activity by AD016: **A), B)** IC_{50} plots and **C), D)** Morrison plots generated using the same data, either with the E_t constrained to 1nM (blue) or unconstrained (red). The K_i and E_t values calculated for each curve are shown. Enzyme and inhibitor were pre-incubated at 22°C for 15 min and reactions were initiated by the addition of Z-FHL substrate to a final concentration of 1 mM. The final enzyme concentration in each reaction was 1 nM for nACE and cACE.

Similarly, AD014 and AD015, also showed irregular IC_{50} curves with Hill coefficient < -1 that could not be explained by tight-binding inhibition models. Consequently, it was not possible to accurately calculate K_i values from IC_{50} values. One way to avoid the complication arising from the effect of substrate competition on inhibition constants is to carry out the assays using substrate concentrations well below the K_m of the substrate ($[S] < \frac{Km}{5}$) so that $IC_{50} \approx K_i$ regardless of the off-rate of the inhibitor, and to lower the enzyme concentration to avoid tight binding conditions. However, this is not practical for these assays and would result in very low signal to noise and/or issues with substrate depletion.

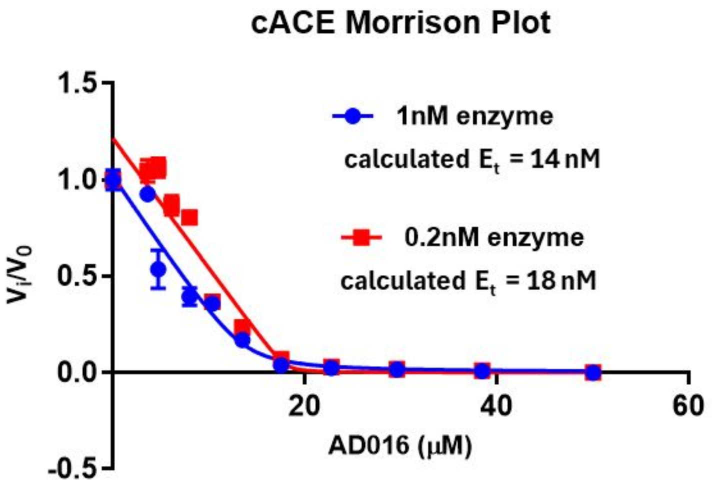

Figure S4: Morrison plots for the inhibition of cACE by compound **AD016**. Two assays with carried out in parallel. One using 1nM final enzyme concentration and the other using 5-fold less (0.2nM). The model was fitted without constraining the $E_{t.}$ The E_t predicted from data set using the Morrison equation is indicated on the graph. A 1.5-fold inhibitor dilution series was used to get more data points on the slope.

Supplementary Methods

Jump dilution nACE and cACE assays

For jump dilution experiments, enzyme and inhibitor were pre-incubated for 15 min and then an aliquot was removed and diluted 20-fold in assay buffer. Activity assays were performed on both the undiluted and diluted enzyme-inhibitor mixes immediately after dilution and ~3h. later. Reaction was initiated with Z-FHL substrate (1mM final concentration). Final enzyme and inhibitor concentrations in reactions were as follows. nACE undiluted samples: 12nM enzyme, 0 - 1μM compound **3b**; nACE diluted samples: 0.6nM enzyme; 0-50 nM compound **3b**; cACE undiluted samples: 3 nM enzyme, 0 – 50 nM compound **3b**; cACE diluted samples: 0.15 nM enzyme; 0 - 2.5 nM compound **3b**. Assays were carried out as described above except the reaction time was increased to 20 min for the diluted samples to ensure sufficient signal was obtained.

Enzyme Data Analysis

Graph-Pad Prism software (San Diego, USA) was used to fit inhibition models to the data using non-linear regression. For the Michaelis-Menten kinetics inhibition assays, fits for competitive, non-competitive and mixed inhibition models were compared in Prism using the F-test to determine the preferred model for each data set.

For IC_{50} inhibition assay, velocity vs Log(inhibitor) curves with variable slope were fitted to the data using this equation. Morison curves were fitted to the same data using this equation:

Y=(1-((((Et+X+(K_i^{app}))-(((Et+X+(K_i^{app}))^2)-4*Et*X)^0.5))/(2*Et)))

Where Y is V_i/V_o and X is inhibitor concentration
